# Double-walled Al-based MOF with large microporous specific surface area for trace benzene adsorption

**DOI:** 10.1038/s41467-024-47612-x

**Published:** 2024-04-13

**Authors:** Laigang Hu, Wenhao Wu, Min Hu, Ling Jiang, Daohui Lin, Jian Wu, Kun Yang

**Affiliations:** 1 Department of Environmental Science, Zhejiang University, Hangzhou, 310058 China; 2 Key Laboratory of Environmental Pollution and Ecological Health of Ministry of Education, Hangzhou, 310058 China; 3 Zhejiang Provincial Key Laboratory of Organic Pollution Process and Control, Hangzhou, 310058 China; 4 ZJU-Hangzhou Global Scientific and Technological Innovation Center, Zhejiang University, Hangzhou, 311215 China

**Keywords:** Metal-organic frameworks, Organic-inorganic nanostructures

## Abstract

Double-walled metal-organic frameworks (MOFs), synthesized using Zn and Co, are potential porous materials for trace benzene adsorption. Aluminum is with low-toxicity and abundance in nature, in comparison with Zn and Co. Therefore, a double-walled Al-based MOF, named as ZJU-520(Al), with large microporous specific surface area of 2235 m^2^ g^–1^, pore size distribution in the range of 9.26–12.99 Å and excellent chemical stability, was synthesized. ZJU-520(Al) is consisted by helical chain of AlO_6_ clusters and 4,6-Di(4-carboxyphenyl)pyrimidine ligands. Trace benzene adsorption of ZJU-520(Al) is up to 5.98 mmol g^–1^ at 298 K and *P/P*_*0*_ = 0.01. Adsorbed benzene molecules are trapped on two types of sites. One (site I) is near the AlO_6_ clusters, another (site II) is near the N atom of ligands, using Grand Canonical Monte Carlo simulations. ZJU-520(Al) can effectively separate trace benzene from mixed vapor flow of benzene and cyclohexane, due to the adsorption affinity of benzene higher than that of cyclohexane. Therefore, ZJU-520(Al) is a potential adsorbent for trace benzene adsorption and benzene/cyclohexane separation.

## Abstract (web summary)

Trace benzene poses a risk to the health and safety of humans, resulting in a challenging task. Here authors synthesise double-walled Al-based MOF ZJU-520(Al) with trace benzene adsorption (5.98 mmol g^–1^) and excellent benzene/cyclohexane separation ability.

## Introduction

Double-walled metal-organic frameworks (MOFs), such as BUT-53(Co) to BUT-57(Co)^1^ and BUT-58(Zn)^1^, with highly customizable and tunable porous structure, exhibit excellent trace adsorption for volatile organic compounds (VOCs) such as benzene due to their unique double-walled structure. For example, BUT-54(Co) showed benzene adsorption amount of 4.31 mmol g^–1^ at *P/P*_*0*_ = 0.01^1^, which is the largest one reported. However, those double-walled MOFs are synthesized using Zn and Co with high price and toxicity. Al element is with low toxicity and abundance in nature^2–4^, compared with Zn and Co. Moreover, the microporous specific surface areas of those reported double-walled MOFs are in the range of 849–1128 m^2^ g^–1 ^^1^, lower than that of reported Al-based MOFs, such as Al-PyrMOF (1592 m^2^ g^–1^)^5^, Al-PMOF (1815 m^2^ g^–1^)^5^ and MOF-519(Al) (2400 m^2^ g^–1^)^6^, limiting its adsorption for VOCs including benzene^7^. Therefore, synthesizing double-walled Al-based MOFs with high microporous specific surface area could be a better strategy for trace adsorption of VOCs including benzene.

Herein, a double-walled Al-based MOF, named as ZJU-520(Al), was synthesized by helical chain of AlO_6_ cluster and 4,6-Di(4-carboxyphenyl)pyrimidine (H_2_DBP) ligands in this study. ZJU-520(Al) is with large microporous specific surface area of 2235 m^2^ g^–1^ and pore size distribution (PSD) in the range of 9.26–12.99 Å. It exhibits thermal and chemical stability: a pH stability between pH 4 and 12 and thermal stability until 250 °C. Most interestingly, it can adsorb trace benzene up to 5.98 mmol g^–1^ at *P/P*_*0*_ = 0.01 and 298 K, and separate trace benzene from mixed vapor flow of benzene and cyclohexane, suggesting that it is a potential adsorbent for trace benzene adsorption/separation.

## Results

### Structural characterization

**Fig. 1:**
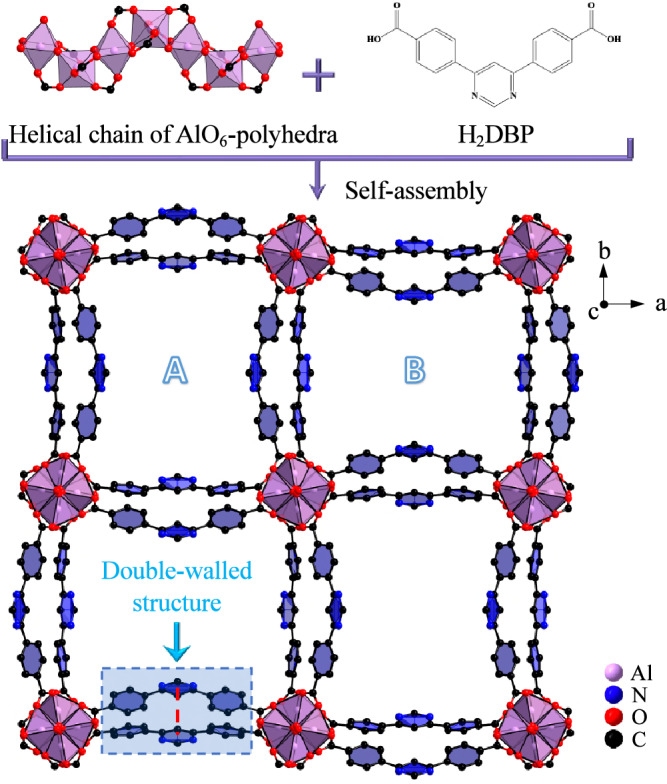
Crystal structure of ZJU-520(Al). Structure description of double-walled ZJU-520(Al), with two types of micropores (named as A and B), consisted by helical chain of AlO_6_-polyhedra and H_2_DBP ligand, as viewed from c axis, and the distance of double-wall on ZJU-520(Al) in red dotted line.

Crystals of ZJU-520(Al) are rod-shaped (Supplementary Fig. 1), with high purity due to the agreement of the theorical powder X-ray diffraction (PXRD) patterns with the experimental PXRD patterns (Supplementary Fig. 2), which can be easily upscaled. ZJU-520(Al) is in the tetragonal crystal system with *I4*_*1*_*md* space group and lattice parameters (a = b = 36.65 Å, c = 10.56 Å), identified by single crystal X-ray diffraction (SC-XRD) analysis (Supplementary Tables 1–3). Each Al^3+^ center, in the helical chain of AlO_6_ cluster, is octahedrally coordinated with O atoms from four DBP^2–^ ligands and two bridging hydroxyl anions (Fig. 1), forming the double-walled structure of ZJU-520(Al) (Fig. 1). The distance of double-wall on ZJU-520(Al) is 3.75 Å (Fig. 1, in red dotted line), slightly higher than that of BUT-53(Co), i.e., 3.00 Å, similar to that of BUT-54(Co) to BUT-58(Zn) (Supplementary Fig. 3)^1^. The AlO_6_ clusters further coordinate to DBP^2–^ ligands, forming the 3D framework (Fig. 1), identified by the slight blue shift of the peaks of carbonyl group at 1589 cm^–1^ in Fourier-transform infrared spectra of ZJU-520(Al) from that of H_2_DBP at 1682 cm^–1^ (Supplementary Fig. 4). ZJU-520(Al) is with two types of 1D channels, named as A with narrow channel and B with wide channel here (Fig. 1), respectively. Those two channels are formed due to the 11.01° rotation of AlO_6_ clusters (Supplementary Fig. 5), in comparison with the AlO_6_ clusters of CAU-10(Al) (Supplementary Fig. 6)^8,9^. Elemental analysis results for activated ZJU-520(Al) (Supplementary Table 4) reveal Al, C, O and N as uniform dispersions, and consistent with its molecular formula [Al(OH)(DBP)], obtained from SC-XRD analysis (Supplementary Table 1), identified by energy dispersive spectroscope (EDS) mapping and EDS linear scans (Supplementary Fig. 7).

### Specific surface area and stability

**Fig. 2:**
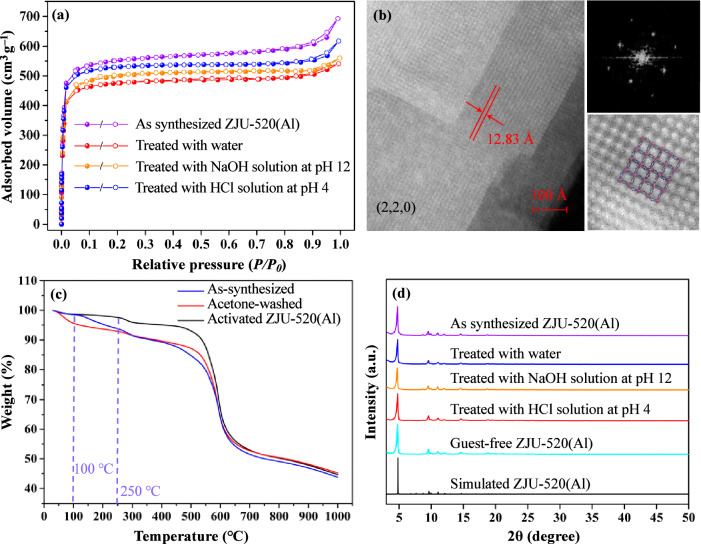
Specific surface area and stability for ZJU-520(Al). N_2_ adsorption-desorption isotherms in filled-white circles, respectively (**a**), HRTEM image taken along the (2, 2, 0) direction (**b**), TGA curves (**c**) and PXRD patterns (**d**) of ZJU-520(Al) with and without treatment.

ZJU-520(Al) exhibits a special surface area (*S*_*BET*_) of 2235 m^2^ g^–1^, derived from the nitrogen (N_2_) adsorption-desorption isotherm at 77 K (Fig. 2a) by Brunauer-Emmett-Teller (BET) method (Supplementary Fig. 8). The experimental *S*_*BET*_ is consistent with the theorical specific surface area of 2348 m^2^ g^–1^, obtained from Grand Canonical Monte Carlo (GCMC) simulation^10–12^, larger than the reported double-walled MOFs^1^ in the range of 849–1128 m^2^ g^–1^ (Supplementary Table 5). For example, the *S*_*BET*_ of double-walled ZJU-520(Al) is 1.98 times that of double-walled BUT-54(Co) (1128 m^2^ g^–1^) and 2.63 times that of double-walled BUT-58(Zn) (849 m^2^ g^–1^)^1^. The total pore volume of activated ZJU-520(Al) is 0.84 cm^3 ^g^–1^ (Supplementary Fig. 9), in agreement with the calculated theorical pore volume of 0.95 cm^3^ g^–1^ by GCMC simulation^13^. ZJU-520(Al) is a microporous material, identified by its N_2_ adsorption-desorption isotherm belonging to type I^9,14,15^ (Fig. 2a) and its PSD in the range of 9.26–12.99 Å with pore dimensions centered at 10.96 Å (Supplementary Fig. 9). It is with two types of microporous channels (Supplementary Fig. 10), using N_2_ as probe^16^ to simulate the pore structure of ZJU-520(Al). One channel, named as A, is with the distance of 10.76–16.07 Å (Supplementary Fig. 11a), while another channel, named as B, is slightly larger with the distance of 11.09–16.67 Å (Supplementary Fig. 11b), in line with the result of PSD (Supplementary Fig. 9). The interplanar spacing is 12.83 Å along the (2, 2, 0) direction, observed from high-resolution transmission electron microscopy (HRTEM) image (Fig. 2b), in agreement with the theorical interplanar spacing of 12.96 Å (Supplementary Table 6). Furthermore, the channel along the (2, 2, 0) direction in the two-dimensional image, calculated by the inverse Fourier transformation from amplitudes extracted from HRTEM image^17,18^, is in good agreement with the framework of ZJU-520(Al) (Fig. 2b).

ZJU-520(Al) is with thermal stability until 250 °C, identified by the thermal gravimetric analysis^12,19^ of activated ZJU-520(Al) (Fig. 2c, in black line) and temperature-variable PXRD patterns^20^ (Supplementary Fig. 12). The quality of activated ZJU-520(Al) is without obvious change until 250 °C due to mostly DMF and water molecules having been removed, and that of acetone-washed ZJU-520(Al) is decreased (Fig. 2c, in red line) below 100 °C due to the evaporation of acetone molecules, and that of as-synthesized ZJU-520(Al) is decreased (Fig. 2c, in blue line) in the range of 100–250 °C due to the evaporation of water and DMF molecules^21,22^. The framework of ZJU-520(Al) also exhibits excellent chemical stability, identified by the N_2_ adsorption-desorption isotherms (Fig. 2a) and PXRD patterns (Fig. 2d) of ZJU-520(Al) without obvious changes, after being treated with water (pH = 7), HCl solution (pH = 4) and NaOH solution (pH = 12). The thermal and chemical stability of ZJU-520(Al) can be attributed to electronic-withdrawing effect of pyrimidine N atoms on H_2_DBP ligands (Supplementary Fig. 13 and Supplementary Table 7) enhancing the Al – O coordinate bond^23,24^.

### Static and dynamic adsorption

**Fig. 3:**
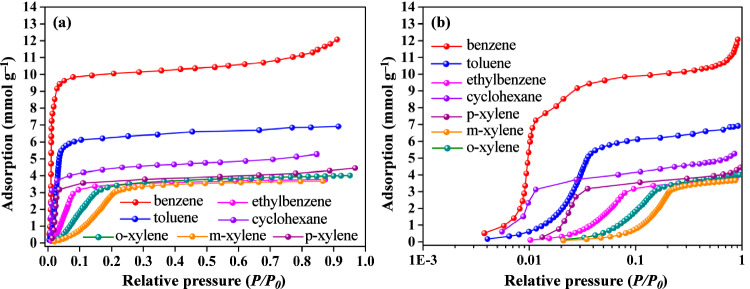
Static adsorption. Adsorption isotherms of BTEX and cyclohexane by ZJU-520(Al) at 298 K with *P/P*_*0*_ at normalized scale (**a**) and logarithmic-scale (**b**).

**Fig. 4:**
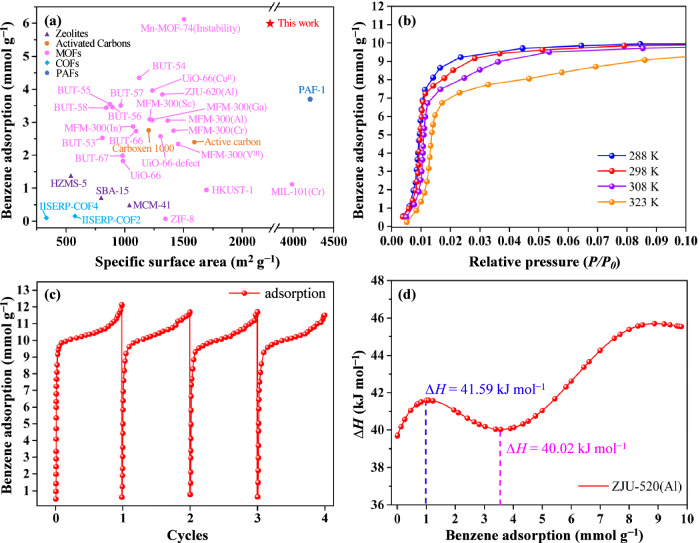
Trace benzene adsorption properties. Adsorption of benzene at 298 K and *P/P*_*0*_ = 0.01 by adsorbents with their specific surface area (**a**), and adsorption isotherms of benzene with *P/P*_*0*_ up to 0.10 at 288, 298, 308 and 328 K (**b**), cyclical benzene adsorption isotherms (**c**) as well as isosteric heat (**d**) of ZJU-520(Al).

The trace concentration of benzene is at the low-pressure (*p* < 1.20 mbar, *P/P*_*0*_ < 0.01)^25^. Therefore, benzene adsorption amount is critically significant at *P/P*_*0*_ = 0.01 for accessing the trace adsorption level. ZJU-520(Al) can adsorb trace benzene up to 5.98 mmol g^–1^ (*Q*_0.01_) at 298 K and *P/P*_*0*_ = 0.01 (Fig. 3a, b and source data), higher than double-walled BUT-54(Co) (*Q*_0.01_ = 4.31 mmol g^–1^)^1^ and other previously reported benzene adsorbents (Fig. 4a and Supplementary Table 5), including PAF-1 (*Q*_0.01_ = 3.65 mmol g^–1^)^26^, ZJU-620(Al) (*Q*_0.01_ = 3.80 mmol g^–1^)^4^ and UiO-66(Cu^II^) (*Q*_0.01_ = 3.92 mmol g^–1^)^25^. Isotherms of benzene adsorption on ZJU-520(Al) are quickly increased at trace concentration and reached a plateau at *P/P*_*0*_ = 0.10 (Fig. 4b), indicating the high affinity between adsorbates and framework^27^. Even at 308 K, ZJU-520(Al) can keep excellent trace benzene adsorption of 3.63 mmol g^–1^ (*Q*_0.01_) and rapidly up to 8.09 mmol g^–1^ at *P/P*_*0*_ = 0.02 (Fig. 4b). It also has excellent saturation adsorption capacity for benzene (12.07 mmol g^–1^), toluene (6.86 mmol g^–1^), ethylbenzene (3.73 mmol g^–1^), ortho-xylene (3.94 mmol g^–1^), meta-xylene (3.65 mmol g^–1^), para-xylene (4.20 mmol g^–1^) and cyclohexane (5.27 mmol g^–1^) at 298 K (Fig. 3a, b). Assuming the void pore volume of activated ZJU-520(Al) is fully occupied by benzene, the calculated maximum benzene adsorption by activated ZJU-520(Al) is 9.44 mmol g^–1^, slightly lower than the experimental benzene adsorption (12.07 mmol g^–1^) at *P/P*_*0*_ = 0.91 due to the condensation of benzene vapor. Besides, ZJU-520(Al) is with excellent regenerate ability, due to benzene adsorption without obvious loss at least 4 times (Fig. 4c) and its framework with integrality due to the PXRD patterns of ZJU-520(Al) without obvious changes (Supplementary Fig. 14) after cyclical benzene adsorption-desorption experiments.

The isosteric heat (*∆H*) values of benzene adsorption on ZJU-520(Al) are modestly high and increased in two steps (Fig. 4d), calculated from the isotherms at four temperatures (Fig. 4b). One step is increased at low benzene adsorption (0.00013–1.10 mmol g^–1^) with the *∆H* values of 39.68–41.59 kJ mol^–1^ owing to the dominance of guest—host interactions^25^. Another step is increased at high benzene adsorption (3.56–9.79 mmol g^–1^) with the *∆H* values of 40.02–45.54 kJ mol^–1^ due to strong guest – guest interactions, such as the packing of benzene molecules^25^. The high *∆H* values indicate that strong interaction with benzene molecules exists even at low concentration, in agreement with its type I benzene adsorption isotherm (Fig. 3a). Moreover, the *∆H* value at benzene loading of 1.00 mmol g^–1^ is 41.59 kJ mol^–1^, close to the other MOFs with excellent trace benzene adsorption, such as UiO-66-defect (47.00 kJ mol^–1^)^25^, MFM-300 (Sc) (42.00 kJ mol^–1^)^25^ and BUT-67(Zr) (55.00 kJ mol^–1^)^26^, indicating that it needs moderate energy for regeneration^28^.

The dynamic benzene adsorption amounts of ZJU-520(Al) at 10 and 1000 ppm are 0.56 and 3.86 mmol g^–1^ (Supplementary Fig. 15a), respectively, using dynamic breakthrough device (Supplementary Fig. 16). The 5% breakthrough time (outlet concentration of benzene reached the 5% of inlet concentration, *t*_*5%*_) of ZJU-520(Al) are 70 and 50 min (Supplementary Fig. 15b), under dry and 30% RH condition, respectively. The *t*_*5%*_ of ZJU-520(Al) at 50% RH is 45 min, close to the value (50 min) at 30% RH (Supplementary Fig. 15b), which can be attributed to ZJU-520(Al) with weak internal hydrophobicity^29^ due to its water adsorption isotherm belonged to type V (Supplementary Fig. 6c). Therefore, the behavior of ZJU-520(Al) adsorbed benzene is affected by water vapor, but this impact is limited.

### Adsorption mechanism

**Fig. 5:**
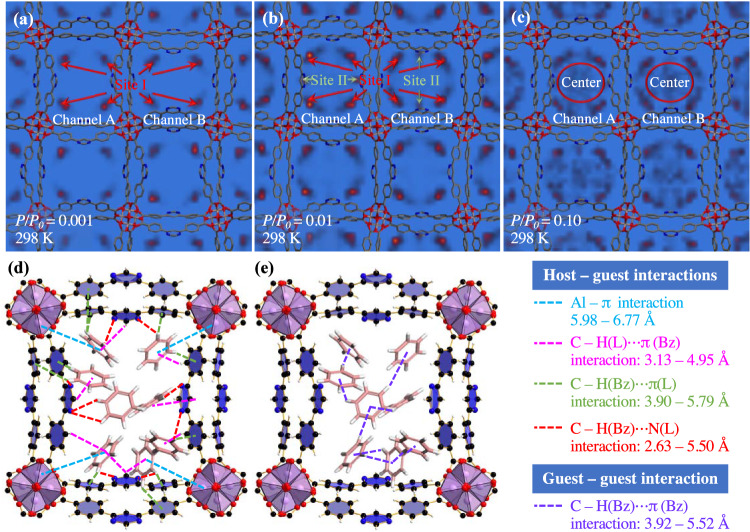
Benzene adsorption mechanism. Potential fields of benzene on ZJU-520(Al) at *P/P*_*0*_ = 0.001 (**a**), 0.01 (**b**) as well as 0.10 (**c**), and interactions, including host – guest interaction (**d**) and guest – guest interaction (**e**).

GCMC simulations are performed to gain insight into the benzene adsorption sites on ZJU-520(Al). Before GCMC simulation, the partial charges of ZJU-520(Al) and adsorbates, such as benzene and toluene, are extracted from density-derived electrostatic and chemical method^30,31^ (Supplementary Fig. 17 and Supplementary Table 8) and restrained electrostatic potential method^4,32^ to account for electrostatic interactions. The simulated parameters of benzene and toluene are listed in Supplementary Table 9. The simulated benzene adsorption isotherm on ZJU-520(Al) is well consistent with the experimental results at low to middle pressures (Supplementary Fig. 18), but slightly smaller than the experimental results at high pressures, due to the condensation of benzene vapor^33^. For example, the theorical benzene adsorption is 6.29 mmol g^–1^ at *P/P*_0_ = 0.01 and 298 K, in agreement with the experimental value (5.98 mmol g^–1^) (Fig. 3a). There exist two types of benzene binding conformations, i.e., site I near the AlO_6_ clusters and site II near the N atom of ligands (Fig. 5a, b and Supplementary Movie 1 for *P/P*_*0*_ = 0.01), due to host – guest interactions (Fig. 4d), identified by the slices of calculated potential field. As is also observed for toluene adsorption (Supplementary Fig. 19 and Supplementary Movie 2). With the *P/P*_*0*_ up to 0.10, benzene molecules also can be adsorbed in the center of channel (Fig. 5c), due to strong guest – guest interactions (Fig. 4d). Benzene molecules, in channel A and B of ZJU-520(Al), preferentially adsorb in site I (Fig. 5a) due to Al – π interactions^34^ and C – H ⋯ X interactions, and then, with the increasing of *P/P*_*0*_, such as up to 0.01, to occupy the site II (Fig. 5b) due to C – H ⋯ N interactions^1,35^. Taking benzene molecules in channel A at *P/P*_*0*_ = 0.01 and 298 K for example, benzene molecules in site I bound to Al atoms of AlO_6_ clusters through Al – π interactions^34^ with the distance of 5.98–6.77 Å (Fig. 5d and Supplementary Fig. 20a, in blue lines), and also bound to the C – H sectors of ligands through C – H(L) ⋯ π(Bz) interactions^36^ with the distance of 3.13–4.95 Å (Fig. 5d and Supplementary Fig. 20b, in pink lines), where H(L) represents the H atom of DBP ligands and Bz represents benzene molecules. Moreover, benzene molecules also could bind to ligands through C – H(Bz) ⋯ π(L) interactions with the distance of 3.90–5.79 Å (Fig. 5d and Supplementary Fig. 20c, in green lines), where H(Bz) represents the H atom of benzene molecules. For site II, benzene molecules bound to N atoms of ligands through C – H(Bz) ⋯ N(L) interactions^1^ with the distance of 2.63–5.50 Å (Fig. 5d and Supplementary Fig. 20d, in red lines), where N(L) represents the N atom of DBP ligands. In addition, benzene molecules in site I and site II are all interacted with other guest molecules, through C – H(Bz) ⋯ π(Bz) interactions with the distance of 3.92–5.52 Å (Fig. 5e and Supplementary Fig. 20e, in purple lines), as is also reported by the previous study^25^. Besides, the simulated benzene adsorption of single-walled ZJU-520(Al) is just 4.45 mmol g^–1^ (Supplementary Fig. 21), that of double-walled ZJU-520(Al) up to 6.29 mmol g^–1^, due to its double-walled structure. Therefore, the host – guest interactions, guest – guest interactions, and double-walled structure drive the benzene adsorption by ZJU-520(Al), even at *P/P*_*0*_ up to 0.01.

The preferential adsorption of benzene molecules in site I (Fig. 5a) also can be explained by the calculated binding energy (–69.68 kJ mol^–1^) between benzene molecules and ZJU-520(Al), which is higher than that in site II (–46.23 kJ mol^–1^). The binding energy is calculated based on the low-energy structure role of ZJU-520(Al) with adsorbed benzene molecules, using the density functional theory calculation method^37–40^. Benzene molecules in site I have stronger charge density than that in site II, making benzene molecules preferentially adsorb in site I rather than site II especially at trace concentration (Fig. 5a, b), due to the electron accumulation^41^ in the center of benzene molecule (site I) and depletion^1^ that of benzene molecule (site II and center) (Supplementary Fig. 22).

### Separation of benzene and cyclohexane

**Fig. 6:**
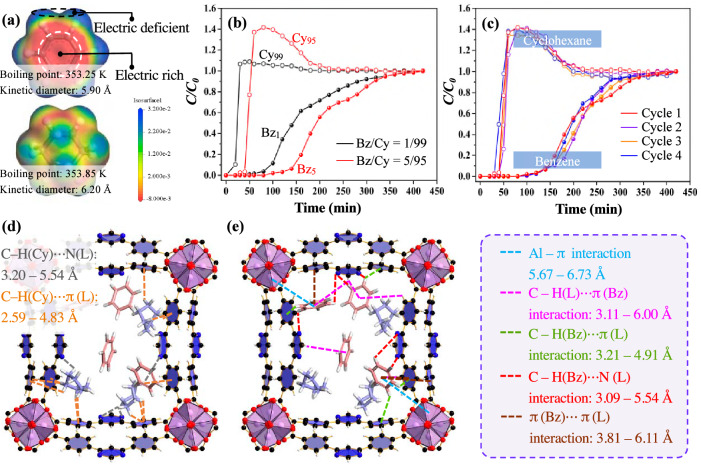
Dynamic adsorption experiments for benzene and cyclohexane. Physical properties of benzene and cyclohexane (**a**), breakthrough curves for benzene/cyclohexane separations (**b**), cyclical benzene/cyclohexane separations (**c**) on ZJU-520(Al), and its interactions with cyclohexane (**d**) and benzene (**e**).

Separating benzene (Bz) from cyclohexane (Cy) is a difficult task in industries such as nylon production^22,42^, due to similar boiling points of Bz (353.25 K) with Cy (353.85 K)^43^ and similar kinetic diameters of Bz (5.90 Å) with Cy (6.20 Å)^44^ (Fig. 6a). The adsorption of ZJU-520(Al) for benzene are 6.19, 3.65 mmol g^–1^ higher than that for cyclohexane at *P/P*_*0*_ = 0.85, 0.01, respectively (Fig. 3 and Supplementary Fig. 23). The ideal adsorbed solution theory (IAST) selectivity value^28,45^ of Bz/Cy mixture at vapor volume of 50/50 by ZJU-520(Al) is 29.86 (Supplementary Fig. 24), exceeding that by CUB-5 (4.2)^46^, MFOF-1 (5.3)^47^ and Mn-TCNQ-bpy (15.2)^47^ (Supplementary Fig. 25). Before IAST selectivity, experimental single-component gas isotherms, including Bz and Cy, are well fitted by Dual-site Langmuir-Freundlich model (Supplementary Fig. 26 and Supplementary Table 10). In the breakthrough experiments of Bz/Cy (5/95, in red line) and Bz/Cy (1/99, in black line) mixture vapor (Fig. 6b), pure cyclohexane elutes with high-purity firstly, whereas trace benzene is still adsorbed in the fixed bed and retained with a longer time course (Fig. 6b and Supplementary Fig. 27), further indicating its excellent trace benzene separation from mixed vapors of Bz/Cy. Taking 5:95 (v/v) Bz/Cy mixture separation for example, cyclohexane molecule first elutes through the fixed bed at 42 min (Fig. 6b, red hollow point), while benzene molecule elutes at 98 min (Fig. 6b, red solid point). ZJU-520(Al) is also with excellent recyclability for Bz/Cy separation at least 4 times (Fig. 6c). Excellent Bz/Cy separation of ZJU-520(Al) could be attributed to the higher adsorption affinity with benzene than cyclohexane molecules, identified by the higher *∆H* of benzene than that of cyclohexane at trace adsorption of benzene and cyclohexane less than 3.89 mmol g^–1^ (Supplementary Fig. 28a), calculated from benzene isotherms (Fig. 4b) and cyclohexane isotherms (Supplementary Fig. 28b) at various temperatures. Using multicomponent GCMC simulations of benzene and cyclohexane adsorptions^48^, the adsorption site of cyclohexane is near the AlO_6_ cluster due to C – H(Cy) ⋯ N(L) interactions with the distance of 3.20–5.54 Å (Fig. 6d and Supplementary Fig. 29a, in black lines) and C – H(Cy) ⋯ π(L) interactions with the distance of 2.59–4.83 Å (Fig. 6d and Supplementary Fig. 29b, in orange lines), where H(Cy) represents the H atom of cyclohexane molecules, while the adsorbed benzene molecules interact with ZJU-520(Al) through multiple interactions, including Al – π interaction with the distance of 5.67–6.73 Å (Fig. 6e and Supplementary Fig. 30a, in blue lines), C – H(L) ⋯ π(Bz) interactions with the distance of 3.11–6.00 Å (Fig. 6e and Supplementary Fig. 30b, in pink lines), C – H(Bz) ⋯ π(L) interactions with the distance of 3.21–4.91 Å (Fig. 6e and Supplementary Fig. 30c, in green lines), C – H(Bz) ⋯ N(L) interactions with the distance of 3.09–5.54 Å (Fig. 6e and Supplementary Fig. 30d, in red lines) and π(Bz) ⋯ π(L) interactions with the distance of 3.81–6.11 Å (Fig. 6e and Supplementary Fig. 30e, in brown lines). Therefore, ZJU-520(Al) exhibits more multiple interactions with benzene than that with cyclohexane, resulting in the excellent Bz/Cy separation of ZJU-520(Al), even at trace benzene concentration.

## Discussion

ZJU-520(Al), as a double-walled Al-based MOF, exhibits large microporous specific surface area of 2235 m^2^ g^–1^, PSD in the range of 9.26–12.99 Å and excellent chemical stability. It is with trace benzene adsorption of 5.98 mmol g^–1^, at 298 K and *P/P*_*0*_ = 0.01, which is the largest one reported. There exist two types of sites for benzene adsorption, site I near the AlO_6_ clusters due to Al – π interactions and site II near the N atom of ligands due to C – H ⋯ X interactions, such as C – H(L) ⋯ π(Bz), C – H(Bz) ⋯ π(L) and C – H(Bz) ⋯ N(L) interactions, using GCMC simulations. ZJU-520(Al) can separate trace benzene from mixed vapor flow of benzene and cyclohexane effectively, due to the higher adsorption affinity of benzene compared to cyclohexane. Furthermore, it is with good recyclability for trace benzene adsorption and separation of benzene/cyclohexane at least 4 times. Therefore, ZJU-520(Al) can be a potential material for trace benzene adsorption and benzene/cyclohexane separation.

## Methods

### Chemicals and materials

All commercial chemicals were purchased without further extra purification. 4,6-Di(4-carboxyphenyl)pyrimidine (H_2_DBP, 98%) was supplied from Extension Technology Co., Ltd., China. Aluminum nitrate nonahydrate (Al(NO_3_)_3_·9H_2_O, 99%), formic acid (99%), *N,N*-dimethylformamide (DMF, 99%), acetone (98%) and KBr (99%) were purchased from Aladdin Reagent Co., Ltd., China. Benzene (>98%), toluene (>98%), ethylbenzene (>98%), ortho-xylene (>98%), meta-xylene (>98%), para-xylene (>98%) and cyclohexane (≥98%) were all supplied from Shanghai Macklin Biochemical Co., Ltd., China.

### Synthesized ZJU-520(Al)

H_2_DBP (22.8 mg, 0.07 mmol), Al(NO_3_)_3_·9H_2_O (30 mg, 0.08 mmol) and 0.02 mL formic acid were mixed and ultrasonically dissolved in 1.5 mL DMF. This mixture was heated at 130 °C for 72 h in the 50 mL Teflon vessel to yield rod-shaped crystalline products after cooling down to room temperature. The as-synthesized products were washed with DMF for three times. Then, the crystals were washed with acetone six times over the course of three days. Finally, the products were dried in the vacuum oven for 24 h at 80 °C (yield: ~10.21 mg). The synthesis scale can be proportionally increased. For example, H_2_DBP (0.32 g, 1.00 mmol) and Al(NO_3_)_3_·9H_2_O (0.56 g, 1.49 mmol) are dissolved in 15 mL DMF, followed 0.20 mL formic acid added into the mixture. Then, the mixture is put into Teflon vessel, and heated at 135 °C for 66 h. The as-synthesized ZJU-520(Al) is washed by DMF and acetone, and dried in the vacuum oven at 80 °C for 24 h. Finally, the yield of ZJU-520(Al) is up to 0.16 g, increasing its synthesis in the quantities.

### N_2_ adsorption-desorption measurements

N_2_ adsorption-desorption isotherm on ZJU-520(Al) was measured by AUTOSORB AS–1 physisorption analyzer (Quantachrome, USA) at 77 K. Before the sorption measurement, the samples were heated at 378 K for 24 h under the vacuum environment. The specific surface area of ZJU-520(Al) was calculated by BET method in the range of 0.001–0.053 (*P/P*_*0*_).

### Thermal gravimetric analysis

As-synthesized ZJU-520(Al) samples are obtained from the centrifugation, and heated at 80 °C. Acetone-washed ZJU-520(Al) samples are obtained from the centrifugation, and heated at 25 °C. Activated ZJU-520(Al) samples are washed with DMF and acetone for three times, respectively, and heated at 105 °C to remove the guest molecules. Therefore, as-synthesized ZJU-520(Al) samples are with DMF and a small amount of water molecules. Acetone-washed ZJU-520(Al) samples are mainly with acetone molecules. Activated ZJU-520(Al) samples are almost without DMF and water molecules. Thermal stability examination of samples was performed on the SDT Q600 (Mettler Toledo, Switzerland) instruction, respectively. In detail, samples were put into the instruction and heated from room temperature to 1000 °C with the rate of 5 °C min^–1^ under N_2_ environment.

### Static adsorption

The vapors adsorption isotherms of benzene, toluene, ethylbenzene, meta-xylene, ortho-xylene, para-xylene (BTEX), cyclohexane, and water were measured by JW-ZQ100 vapor adsorption instrument (Beijing JWGB Sci.& Tech. Co., Ltd, China). The vapors were generated by steam generation unit and heating unit. Before the measurement, adsorbate liquids were purified at 77 K in the liquid nitrogen, respectively.

### Dynamic breakthrough experiment

The activated ZJU-520(Al) about 0.10 g was mixed with quartz wool uniformly and put into the fixed bed (6.00 mm outer diameter, 4.00 mm inner diameter, 130.00 mm length) for dynamic breakthrough experiment of benzene/cyclohexane (Bz/Cy) at 298 K, using multi-constituent adsorption breakthrough curve analyzer (BSD-MAB, BSD Instrument Co., Ltd., China). The gas flow of high-purity N_2_ gas (99.99%) was chosen to control the concentration of benzene vapor by bubbling the high-purity benzene liquid (≥99.9%, standard for GC), using mass flow controller (MFC 1). Another high-purity N_2_ gas was used to bubble the high-purity cyclohexane liquid (≥99.9%, standard for GC) to control the concentration of cyclohexane vapor by MFC 2. The entire flow rate was 30 mL min^–1^, controlled by MFC 3. The concentrations of mixture gases (Bz/Cy) were detected by gas chromatography (GC 9790Plus, FULI Instruments Co., Ltd., China) at the fixed inlet and outlet. Furthermore, the points of breakthrough curve were obtained by continuously determining and recording the outlet concentrations at the time interval. In detail, dynamic breakthrough experiments of Bz/Cy (5/95) and Bz/Cy (1/99), with trace benzene concentration, are performed.

### Reporting summary

Further information on research design is available in the Nature Portfolio Reporting Summary linked to this article.

### Supplementary information

Supplementary Information

Peer Review File

Description of Additional Supplementary Files

Supplementary Movie 1

Supplementary Movie 2

Reporting Summary

### Source data

Source Data

## Data Availability

The static adsorption data of ZJU-520(Al) (Fig. 3) has been deposited. Source data are provided with this paper.
