# Supplementary material for: Double-walled Al-based MOF with large microporous specific surface area for trace benzene adsorption
Source: Nat Commun. 2024 Apr 13;15:3204. doi: 10.1038/s41467-024-47612-x (PMC11016061; doi:10.1038/s41467-024-47612-x)
Supplement: Supplementary file 1 — Supplementary Information [file 41467_2024_47612_MOESM1_ESM.pdf]

Supplementary Information

Double-walled Al-based MOF with large microporous specific surface area for trace benzene adsorption

Laigang Hu [a,c,d], Wenhao Wu [a,c,d], Min Hu [a,c,d], Ling Jiang [a,c,d],Daohui Lin [a,c,d], Jian Wu [a,c,d], Kun Yang [a,b,c,d*]

[a] Department of Environmental Science, Zhejiang University, Hangzhou 310058, China;

[b] ZJU-Hangzhou Global Scientific and Technological Innovation Center, Zhejiang University, Hangzhou 311215, China;

[c] Key Laboratory of Environmental Pollution and Ecological Health of Ministry of Education, Hangzhou 310058, China;

[d] Zhejiang Provincial Key Laboratory of Organic Pollution Process and Control, Hangzhou 310058, China;

[*] Corresponding author (Kun Yang): Tel.: 86-571-88982589; Fax: 86-571-88982590;

E-mail: kyang@zju.edu.cn

Supplementary Notes

Synthesized CAU-10(Al)

CAU-10(Al) was synthesized according to previous study [1]. In detail, 1,3-H_2BDC (200 mg, 1.20 mmol) and $Al_2(SO_4)_3 \cdot 18H_2O$ (800 mg, 1.20 mmol) were dissolved in the mixture of 1.00 mL DMF and 4.00 mL. Then, the mixture was added into Teflon vessel and heated at 135 °C for 24 h. The product of CAU-10(Al) was collected by centrifugation, and washed with methanol.

The topology, specific surface area, benzene adsorption, internal/exterior hydrophilicity and hydrophobicity of ZJU-520(Al) and CAU-10(Al) are compared in this work. In detail, the topology of CAU-10(Al) and ZJU-520(Al) are all **yfm**. The experimental specific surface area of CAU-10(Al) is 583.45 $m^2\ g^{-1}$, calculated from 77 K N_2 adsorption-desorption (Supplementary Fig. 6a), lower than that of ZJU-520(Al) (2235 $m^2\ g^{-1}$). The saturation benzene adsorption capacity of ZJU-520(Al) is 12.07 mmol g^{-1}, which is 6.07 times that of CAU-10(Al) (1.98 mmol g^{-1}) (Supplementary Fig. 6b) at 298 K. Both Al-based MOFs are all with weak internal hydrophobicity, due to their water vapor adsorption isotherms (Supplementary Fig. 6c) belonged to type V [2]. The static water contact angle (WCA) of ZJU-520(Al) and CAU-10(Al) are all close to 0° (Supplementary Fig. 6d,e), suggesting their exterior hydrophilicity.

N_2 adsorption-desorption measurements

The pore size distribution (PSD) of ZJU-520(Al) was calculated according to the nonlocal density functional theory (NLDFT) model using the geometry of slit [3]. The

total pore volume of sample was obtained from N_2 adsorption-desorption isotherm at 77 K and relative pressure (P/P_0) of 0.99.

SEM image, EDS mapping and elemental analysis

Scanning electron microscopy (SEM) images and energy dispersive spectroscope (EDS) mapping were taken using a Zeiss GIMINI 300 (Germany). Prior to measurement, samples were coated with Pt to ~ 9 nm thickness in MCIOOO ion sputter (HITACHI, Japan). The C, N, H contents of ZJU-520(Al) are obtained from elemental analyzer (Vario MAX cube, Germany), and Al content is measured by S8 Tiger X-ray Fluorescence Spectrometer (Bruker, Germany). The O content of ZJU-520(Al) is obtained by subtracting the content of Al, C, H, and N from 100%.

Fourier-transform infrared (FT-IR) spectra analysis

The functionalized groups of ZJU-520(Al) and H_2DBP were collected from 400 ~ 4000 cm^{-1} on Bruker-vector-22 by Fourier-transform infrared spectra method. The samples of ZJU-520(Al) (1.00 mg) and H_2DBP (1.05 mg) were mixed with KBr using the agate mortar, then ground and compressed into circular flakes to FT-IR analysis [4], respectively.

Static adsorption of BTEX, cyclohexane and water vapor

The static adsorption of vapors was measured by JW-ZQ100 vapor adsorption instrument equipped with steam generation unit and heating unit. The heating unit of instrument was utilized to maintain the temperature of steam generation unit at 313 K, ensuring the consistent and continuous production of steam [5]. The temperature of

adsorbate samples was kept in the adsorption process, using the heating in water bath methods. Before the measurement, the samples were outgassed at 378 K (105 °C) for 24 h to remove the guest molecules in the pore structure of adsorbent.

To calculate the benzene/cyclohexane (Bz/Cy) separation of ZJU-520(Al), the Dual-site Langmuir-Freundlich (DSLF) model [6], Equation (S1), was chosen to fit the single-component adsorption isotherms of benzene and cyclohexane at 298 K.

$$Q_m = Q_{m1}\frac{b_1 p^n}{1 + b_1 p^n} + Q_{m2}\frac{b_2 p^m}{1 + b_2 p^m} \tag{S1}$$

Where Q_m is the adsorbed amount, mmol g^{-1}; $Q_{mi\,(i=1,2)}$ is the saturation adsorption capacity, mmol g^{-1}; p is the equilibrium pressure of gas, kPa; $b_{i(i=1,2)}$ is the affinity coefficient, kPa^{-1}; n and m are the deviations.

Adsorption selectivity for Bz/Cy gas mixture on ZJU-520(Al) at 298 K was calculated by ideal adsorbed solution theory (IAST) [7].

$$S = \frac{x_1/y_1}{x_2/y_2} \tag{S2}$$

Where x_i and y_i are the molar fractions of components in the adsorbed and bulk phase.

We assume that the pore volume of activated ZJU-520(Al) is fully occupied by adsorbents, such as benzene and cyclohexane. The maximum benzene and cyclohexane adsorption by ZJU-520(Al) are predicted by the following Equation (S3):

$$Q_{max} = \rho_{adsorbate} \times PV \div M \times 1000 \tag{S3}$$

Where ρ is the density of adsorbates, such as benzene (0.8765 g cm^{-3}) and cyclohexane (0.7739 g cm^{-3}); PV is the void pore volume of adsorbent, cm^3 g^{-1}; M is the molecular weight, g mol^{-1}; Q_{max} is the calculated maximum adsorbate adsorption, mmol g^{-1}.

Isosteric heat of adsorption

Isosteric heat of adsorption on ZJU-520(Al) was calculated from Clausius-Clapeyron equation [8] to predict the interaction between adsorbates and adsorbent, using the adsorption isotherms of adsorbates at 288, 298 and 308 K.

$$\text{In}\, P = -\frac{\Delta H_s}{R \times T} + C \tag{S4}$$

Where P is the pressure; R is the ideal gas constant, 8.314 kJ mol^{-1}; C is the constant; T is the temperature, K; ΔH_s is calculated from slopes of plots of In P *versus* $1/T$ of fixed loading.

Dynamic breakthrough experiment

Humid breakthrough experiment of ZJU-520(Al) is also performed. The activated ZJU-520(Al) (200 mg) is also put into the fixed bed. The first N_2 gas is used to bubble the benzene liquid. The water vapor is generated by bubbling N_2 through deionized water. The humidity of gaseous mixture is detected by hygrometer in the mixing vessel. The total gas flow rate is controlled at 30 mL min^{-1}. The outlet concentration of benzene

in the humid/dry breakthrough curves, such as 10 and 1000 ppm, are detected by gas chromatography at the same time interval.

The saturated adsorption capacities of benzene for adsorbents were calculated via the integral of the breakthrough curves, respectively, as shown in Equation (S5).

$$Q = \frac{F \times 10^{-6}}{m} \times \left(C_0 \times \mathrm{t} - \int_0^t C_t dt \right) \quad \text{(S5)}$$

Where Q is the equilibrium adsorption capacity, mg g^{-1}; F is the total flow rate of the gas, mL min^{-1}; m is the mass of the adsorbent, g; C_0 is the initial concentration of benzene at the inlet, mg m^{-3}; C_t is the benzene concentration at the outlet after adsorption for t minutes, mg m^{-3}; t is the adsorption time, min.

Powder X-ray diffraction (PXRD) analysis.

PXRD patterns of ZJU-520(Al) were collected on the diffractometer (D8 ADVANCE, Bruker, Germany) equipped with Cu sealed tube (CuK$\boldsymbol{\alpha}$1 radiation, $\boldsymbol{\lambda}$=1.5418 Å) at 40 kV and 40 mA to characterize crystallinity of synthesized materials. The intensity data of samples was collected from 2° to 50° with a scan step size of 0.02°. The refinement of ZJU-520(Al) structure was performed on the software of GASA II [9].

Single Crystal X-ray diffraction analysis

Single Crystal X-ray diffraction (SC-XRD) data of as-synthesized ZJU-520(Al) crystal, with crystal size of 0.10 × 0.02 × 0.02 mm (Supplementary Fig. 1), was collected at 100.15 K using the Brucker D8 Venture diffractor equipped with the PHOTON II detector, Cryostream 80–400 K (Oxford Cryosystems, United Kingdom), and CuK$\boldsymbol{\alpha}$ ($\boldsymbol{\lambda}$=1.54178 Å) *IμS* microfocus X-ray source with MX Optics and a Kappa

geometry goniometer. The rod-shaped crystal was obtained from mother solution, mounted on the MicroMesh (MiTeGen) with paratone oil. The space group of ZJU-520(Al) belonged to $I4_1md$, solved by SIR2008 [10, 11]. Moreover, the structure of ZJU-520(Al) was determined by intrinsic phasing (SHELXT [12]) and refined by full-matrix least-squares refinement on F^2 (SHELXL) using Olex2 software package [13, 14]. The crystal data of ZJU-520(Al) without guest molecules are obtained from the ZJU-520(Al) with guest molecules (as-synthesized ZJU-520(Al)) by removing the guest solvent molecules, using PLATON SQUEEZE program [15, 16]. Refinement results and details of collected data on ZJU-520(Al) with and without solvent molecules were summarized in Supplementary Table 1, obtained from Olex2 software package. In addition, the selected bond length and angle were also summarized in Supplementary Table 2–3. The SC-XRD data of ZJU-520(Al), containing the crystallographic data for this paper, was deposited in the Cambridge Crystallographic Data Centre (CCDC) database under deposition number CCDC 2288227.

Molecular simulations

Grand Canonical Monte Carlo (GCMC) simulations were carried out on the software of RASPA 2.0 [17] to investigate the vapor adsorption process on ZJU-520(Al). Before GCMC simulations, Helium (He) void fraction (HVF) of ZJU-520(Al) was calculated (ca. 0.64), using He as the probe according to Widom insertions method [17, 18]. Crystal structure of ZJU-520(Al), obtained from SC-XRD analysis, was without further geometry optimization for related simulations and kept rigid during the process

of simulation. The theorical specific surface area and pore volume of ZJU-520(Al) were also obtained from GCMC simulations, using nitrogen (N_2) as probe (1.84 Å) at 77 K [19]. Each point, in simulated adsorption isotherms, consisted of 10,000 initialization cycles and 10,000 production cycles. The adsorbate molecules could be translation, rotation, insertion and deletion in the framework of ZJU-520(Al). The interaction energies, such as van der Waals interaction, between adsorbate-framework were calculated through Lennard-Jones (LJ) potential [20], using the 12.5 Å cutoff to handle the nonbonding interactions [7, 21]. The adsorption temperature was set as 298 K. Furthermore, the density-derived electrostatic and chemical (DDEC) charges of ZJU-520(Al) with period framework were calculated through periodic density functional theory (DFT) methods, using Chargemol software [22, 23]. Restrained electrostatic potential (RESP) charges [24, 25] of adsorbates, such as benzene and cyclohexane, are calculated. Ewald & Group summation method was used to calculate the electrostatic interactions between adsorbate-framework. Universal force-field [26] and Dreiding force-field [27] were used to describe the LJ parameters of framework metal and nonmetal atoms, respectively. For adsorbate molecules, the LJ parameters were obtained from the TraPPE force-field [28]. The 4×4×4 supercells of ZJU-520(Al) were used to simulation adsorption at 298 K. The calculated excess adsorption of adsorbent compared with the experimental adsorption, was obtained from Equation (S6) [29] [30].

$$N_{exc}=N_{abs}-V_p\times\rho_{bulk} \quad \text{(S6)}$$

Where N_{abs} is the absolute adsorption, containing the amount of gas adsorbed in the pore volume in the presence of pore walls, mmol g^{-1}; N_{exc} is the excess adsorption capacity, mmol g^{-1}; V_p is the pore volume of adsorbent, cm^3 g^{-1}; ρ_{bulk} is the bulk density of adsorbate molecule, g cm^{-3}.

Besides, the single-walled ZJU-520(Al) is built by removing half of ligand of double-walled ZJU-520(Al). The open metal sites (OMS) of single-walled ZJU-520(Al) is sealed by C atoms to avoid the function of OMS for benzene adsorption [31], listed in Supplementary Fig. 21. GCMC simulation of single-walled ZJU-520(Al) is in the same condition as double-walled ZJU-520(Al).

Density functional theory (DFT) calculations

Density functional theory (DFT) calculations were implemented for investigating the adsorption energy and electronic density of benzene molecule and ZJU-520(Al) with and without benzene, after optimization structure, using the software of CP2K [32, 33]. The input files of CP2K calculation tasks were generated by Multiwfn software [34]. In detail, the theoretical method of Perdew, Burke and Ernzerhof (PBE) exchange-correlation function was applied with Generalized Gradient Approximation (GGA) [35-37]. The plane wave cutoff energy of this system was set to 400 Ry, with the relative cutoff of 50 Ry and progression factor of 3, according to the previous study [38, 39]. DFT-D3 scheme with semiempirical dispersion corrections [40, 41] was used to compensate the weak interaction, such as the long-range van der Waals dispersion interaction [42] between adsorbate and the skeleton of ZJU-520(Al). The norm-conserving

Goedecker–Teter–Hutter (GTH) pseudopotential [43] and double-ζ valence polarized basis set (DZVP-MOLOPT-SR-GTH) [44, 45] were employed for DFT calculation. The binding energy of adsorbed benzene on ZJU-520(Al) was calculated, according to Equation (S7) [46]. The isosurfaces of charge density difference on ZJU-520(Al) with benzene molecules in site I, site II and center, were calculated by Equation (S8) [47].

$$\Delta \boldsymbol{E}_{ads} = \boldsymbol{E}_{Benzene@ZJU\text{-}520(Al)} - (\boldsymbol{E}_{Benzene} + \boldsymbol{E}_{ZJU\text{-}520(Al)}) \tag{S7}$$

Where $E_{Benzene@ZJU\text{-}520(Al)}$ is the total energy of ZJU-520(Al) with adsorbed benzene molecule in the equilibrium state, kJ mol^{-1}; $E_{Benzene}$ is the energy of benzene, kJ mol^{-1}; $E_{ZJU\text{-}520(Al)}$ is the energy of ZJU-520(Al) without adsorbed benzene molecules, kJ mol^{-1}; ΔE_{ads} is the binding energy of adsorbed benzene molecule on ZJU-520(Al).

$$\Delta \boldsymbol{\rho} = \boldsymbol{\rho}_{Benzene@ZJU\text{-}520(Al)} - (\boldsymbol{\rho}_{Benzene} + \boldsymbol{\rho}_{ZJU\text{-}520(Al)}) \tag{S8}$$

Where $\rho_{Benzene@ZJU\text{-}520(Al)}$ is the charge density of ZJU-520(Al) with benzene, such as benzene molecule in site I, site II and center; $\rho_{Benzene}$ is the charge density of benzene; $\rho_{ZJU\text{-}520(Al)}$ is the charge density of ZJU-520(Al) without benzene; $\Delta\rho$ is the charge density difference of ZJU-520(Al) with benzene.

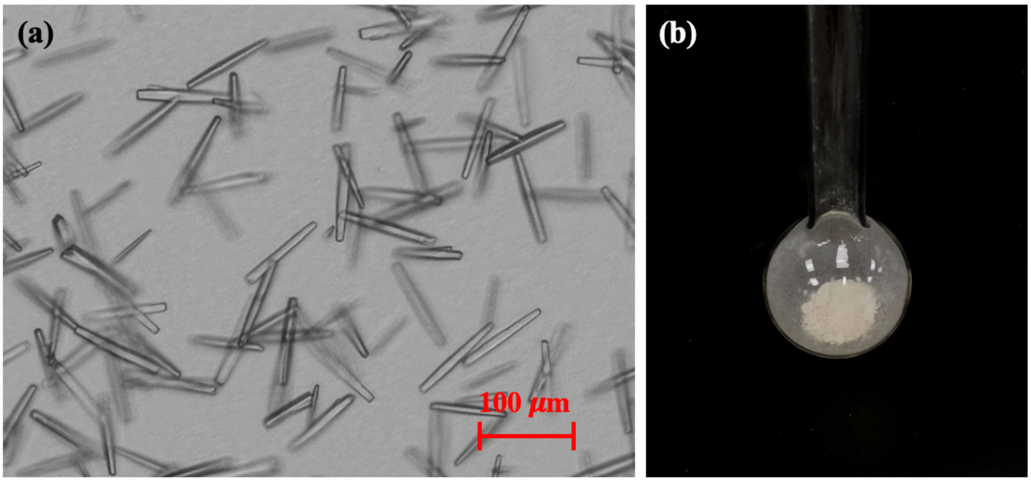

Supplementary Fig. 1 Optical photo of ZJU-520(Al) crystals (a) and white powder (b).

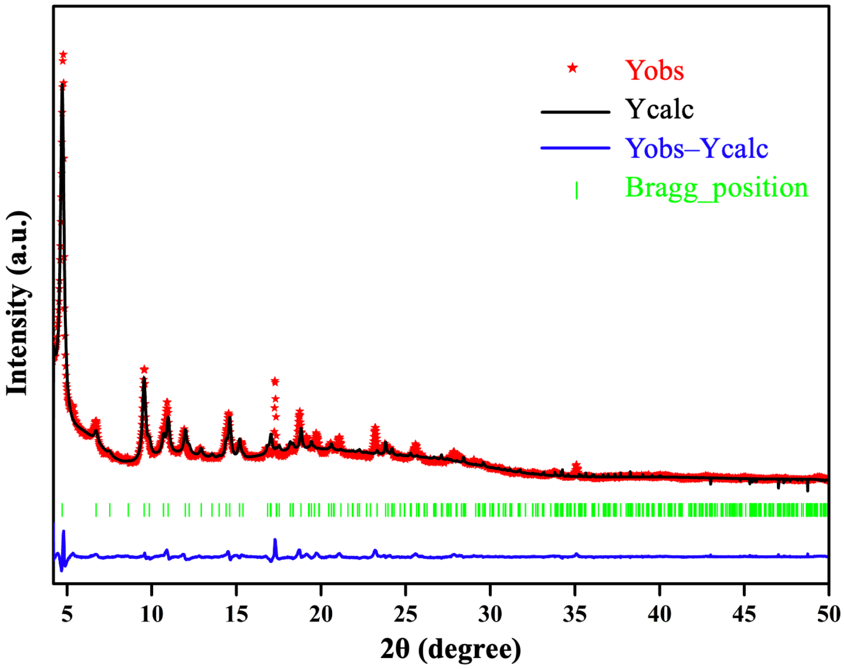

Supplementary Fig. 2 Experimental PXRD patterns of as-synthesized ZJU-520(Al) compared with simulated ZJU-520(Al) diffraction pattern (λ = 1.5418 Å). Observed (Y_{obs}, in red point) and calculated (Y_{calc}, in black line) PXRD patterns and different plot (in blue line) for the Rietveld refinement of ZJU-520(Al). The green vertical bars correspond to the position of Bragg peaks.

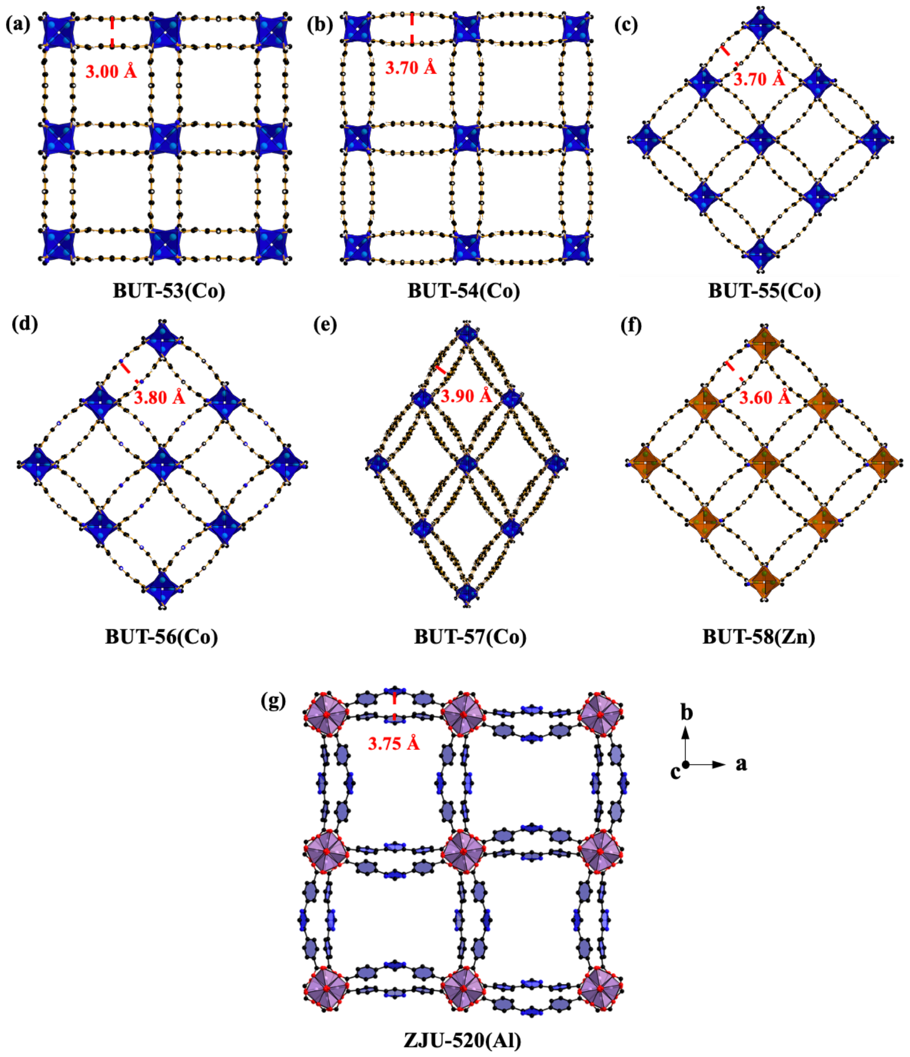

Supplementary Fig. 3 The distance of double-wall on BUT-53 to BUT-58 and ZJU-520(Al), as viewed from c axis.

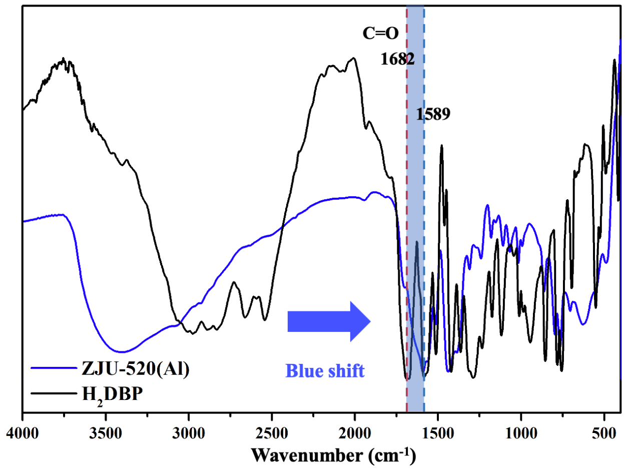

Supplementary Fig. 4 FT-IR spectra of activated ZJU-520(Al) (in blue line) and H_2DBP ligand (in black line).

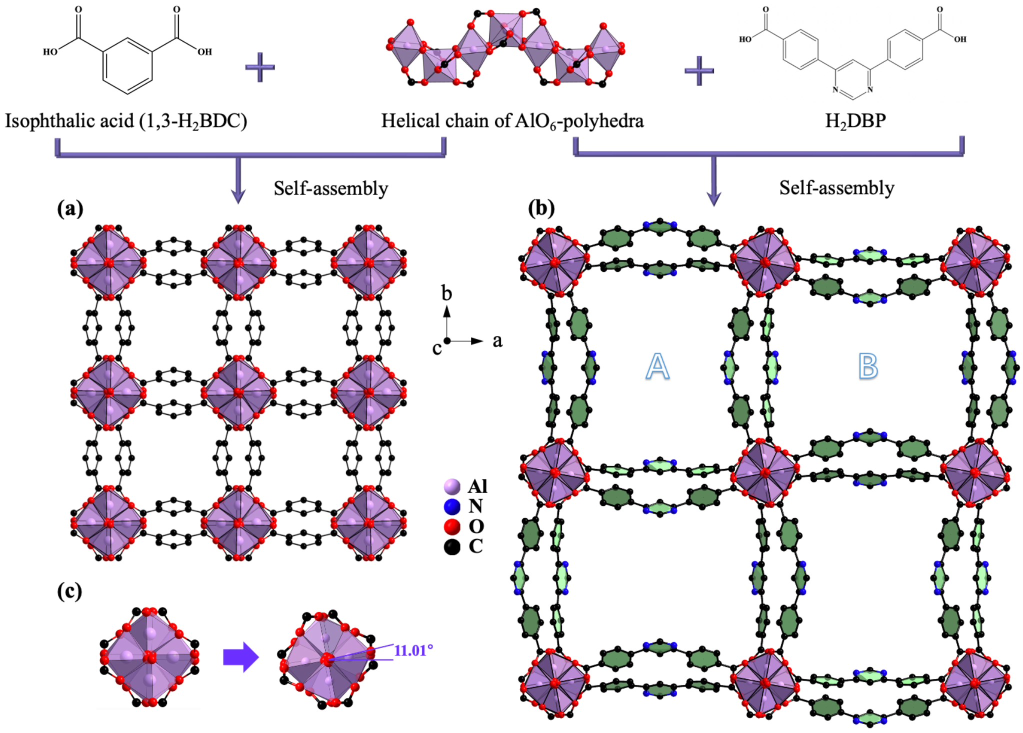

Supplementary Fig. 5 Structure description of CAU-10(Al) (a) and ZJU-520(Al) (b). The AlO_6 cluster of ZJU-520(Al) with the rotation about 11.01 degree (c), compared with that of CAU-10(Al), as viewed from c axis.

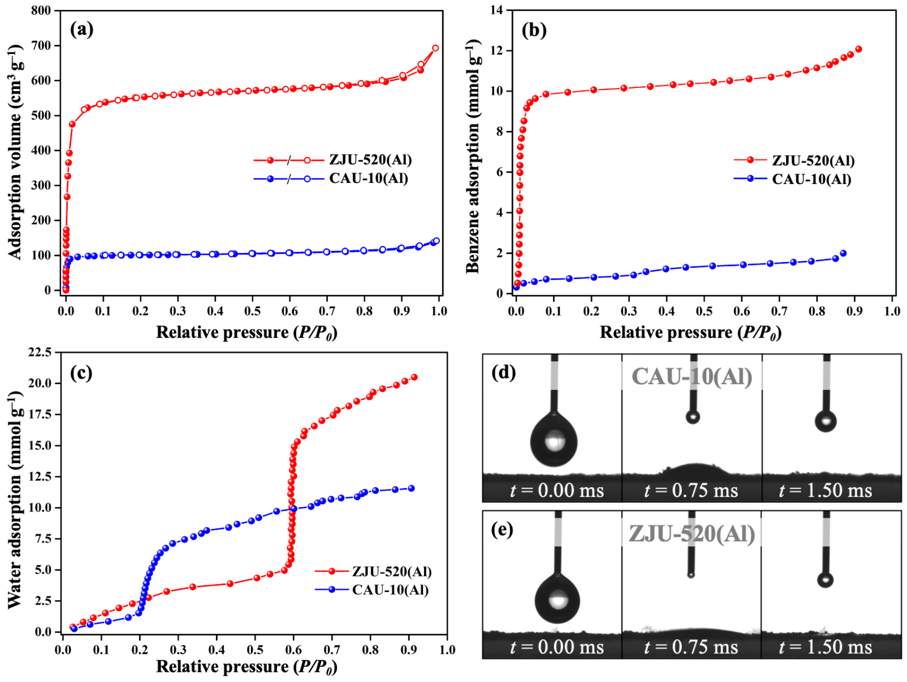

Supplementary Fig. 6 N_2 adsorption-desorption isotherms at 77 K (a), benzene adsorption isotherms (b) at 298 K and water adsorption isotherms (c) at 298 K, and water contact angle of CAU-10(Al) (d) and ZJU-520(Al) (e).

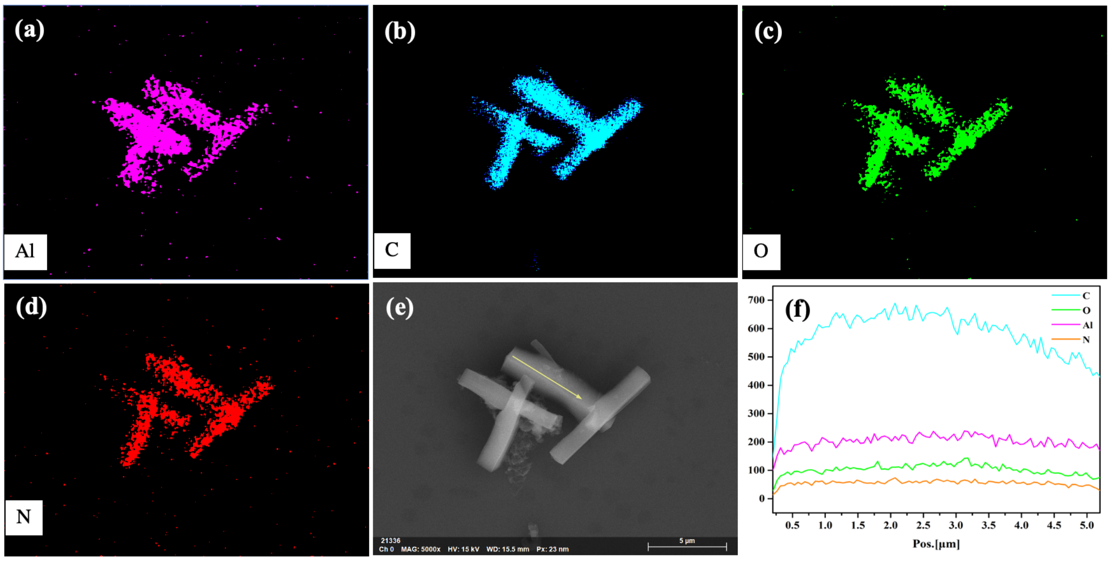

Supplementary Fig. 7 Elemental energy dispersive spectroscope (EDS) spectral mapping, including Al (a), C (b), O (c) and N (d), SEM image (e), EDS linear scans (f) of ZJU-520(Al).

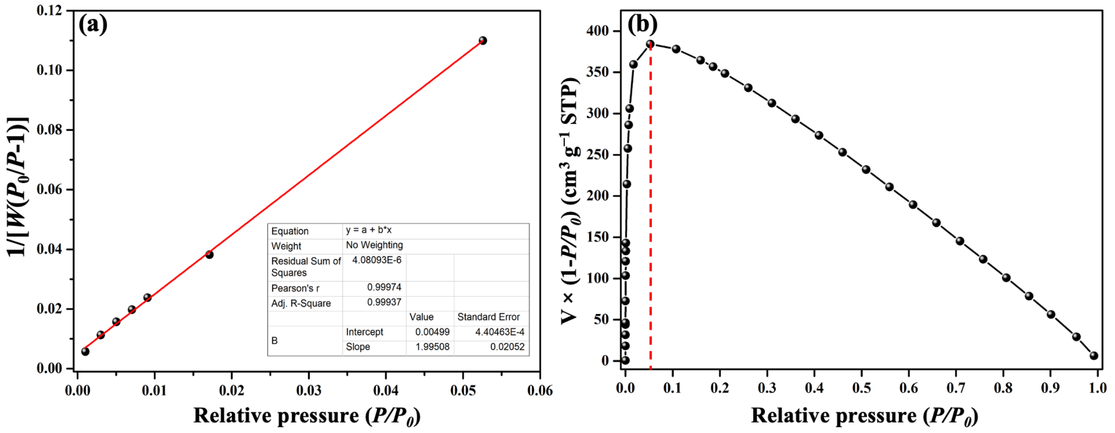

Supplementary Fig. 8 Linear Brunauer-Emmett-Teller (BET) plot (a) and Rouquerol plot (b), *i.e.*, $V\times(1\text{-}P/P_0)$ *vs* P/P_0, of ZJU-520(Al) from N_2 adsorption-desorption isotherm. The specific surface area of ZJU-520(Al) was calculated by BET method in the range of 0.001 – 0.053.

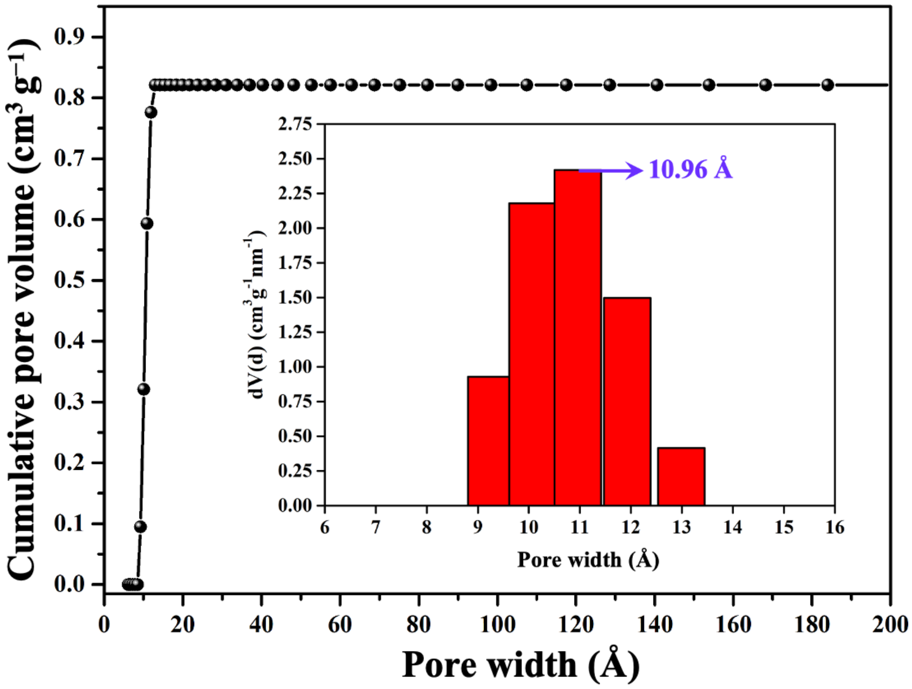

Supplementary Fig. 9 Cumulative pore volume curve and inset PSD plot of ZJU-520(Al).

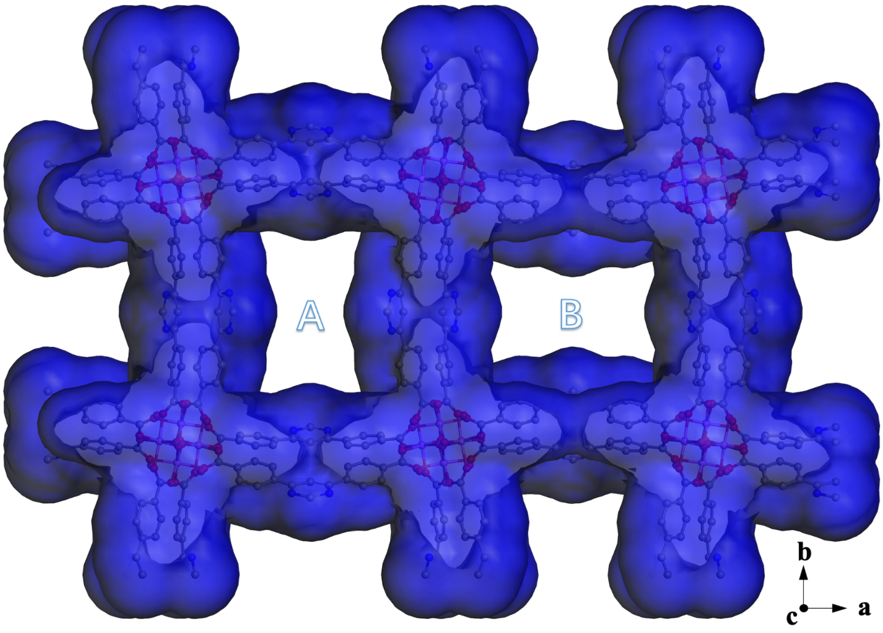

Supplementary Fig. 10 Two types of microporous channels, named as A and B, using N_2 as probe, as viewed from c axis.

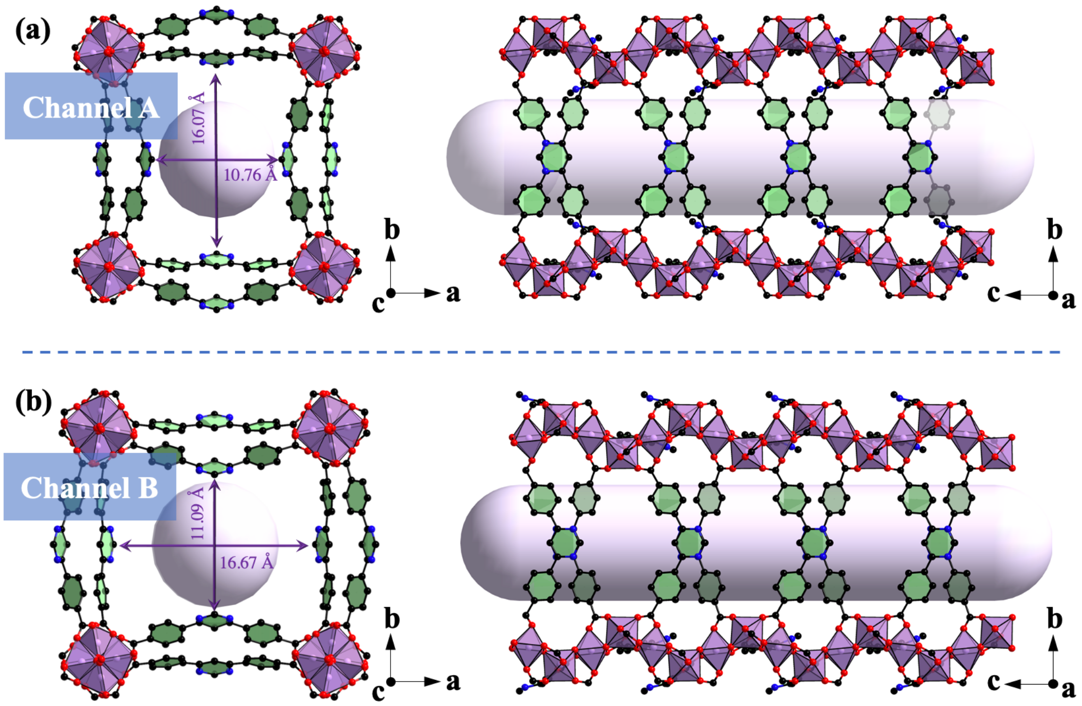

Supplementary Fig. 11 Pore structures of ZJU-520(Al), including channel A (a) and B (b).

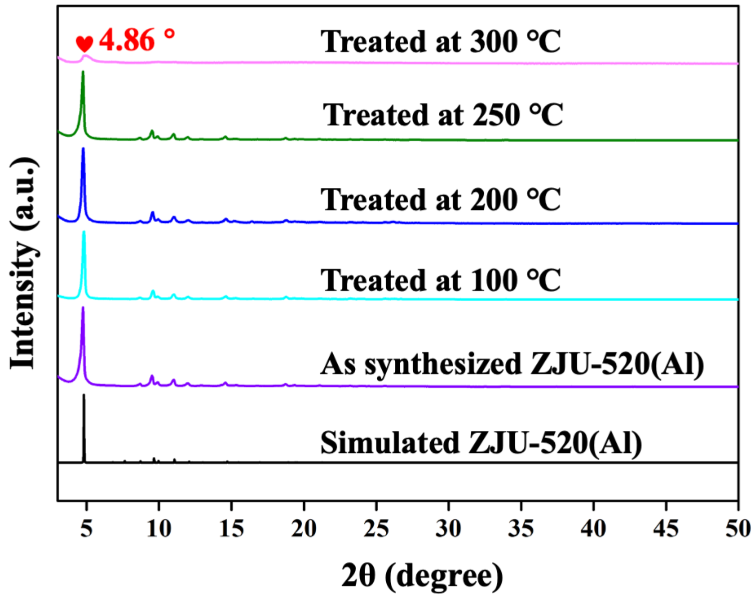

Supplementary Fig. 12 Temperature-variable PXRD patterns of ZJU-520(Al) at 100, 200, 250 and 300 °C.

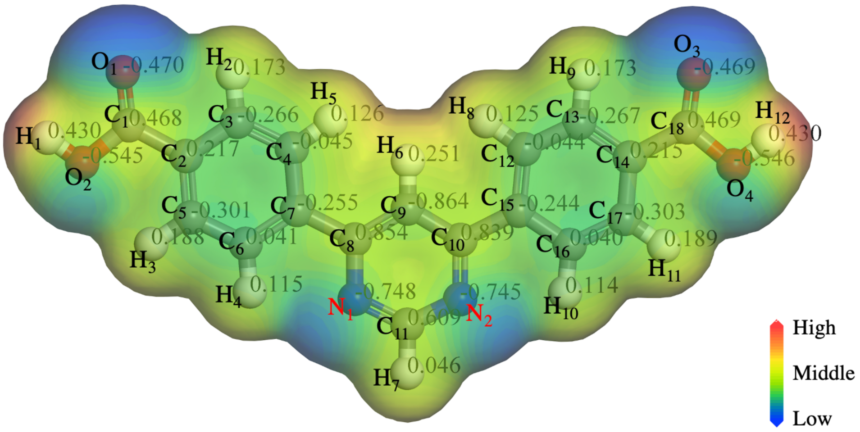

Supplementary Fig. 13 Restrained electrostatic potential (RESP) charge distributions of H_2DBP ligand.

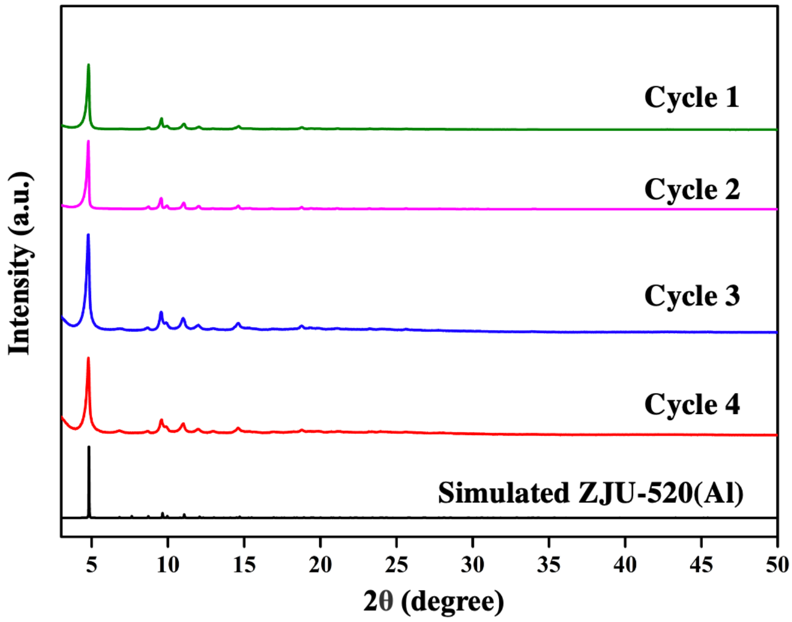

Supplementary Fig. 14 PXRD patterns of ZJU-520(Al) after cyclical benzene adsorption experiments, in comparison with simulated PXRD patterns of ZJU-520(Al), using the same samples for benzene adsorption after degassed at 378 K for 4 hours.

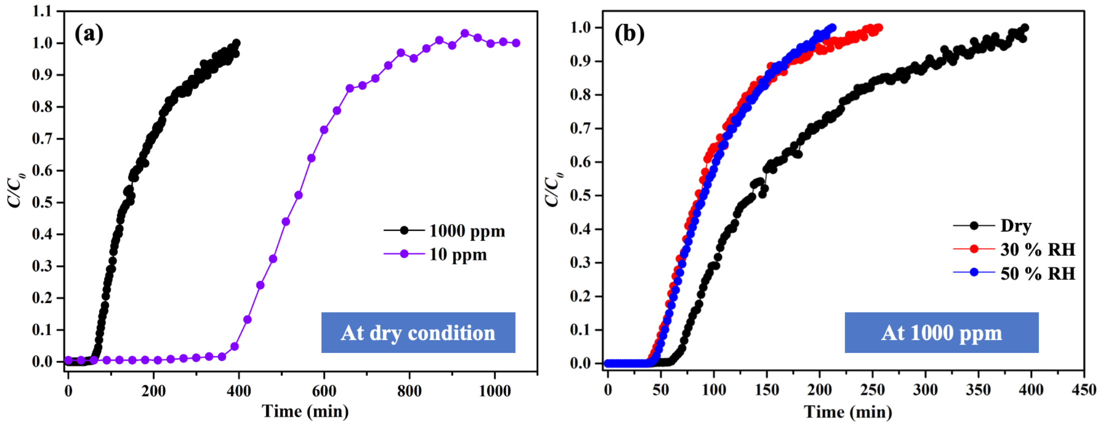

Supplementary Fig. 15 Breakthrough curves of 10, 1000 ppm benzene on ZJU-520(Al) (a) and 1000 ppm benzene at dry, 30% and 50% RH (b).

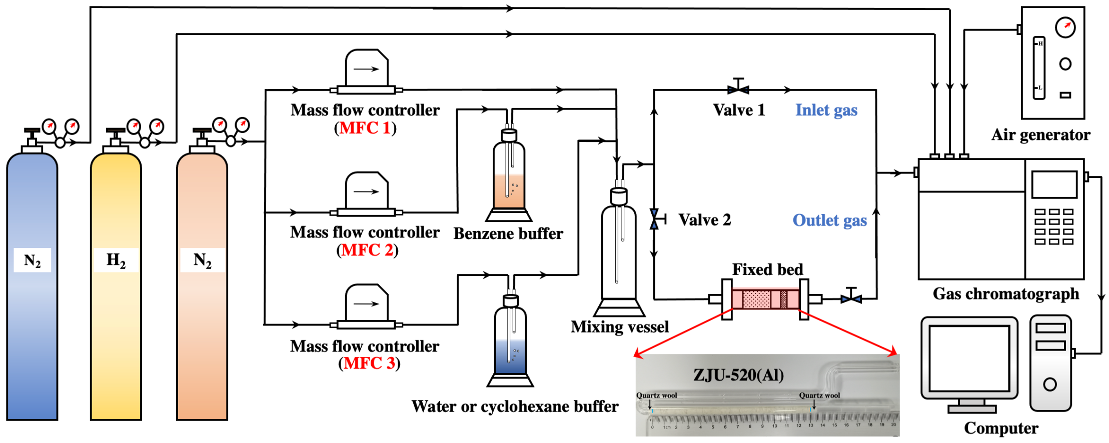

Supplementary Fig. 16 Schematic illustration of dynamic breakthrough experiments of benzene/cyclohexane.

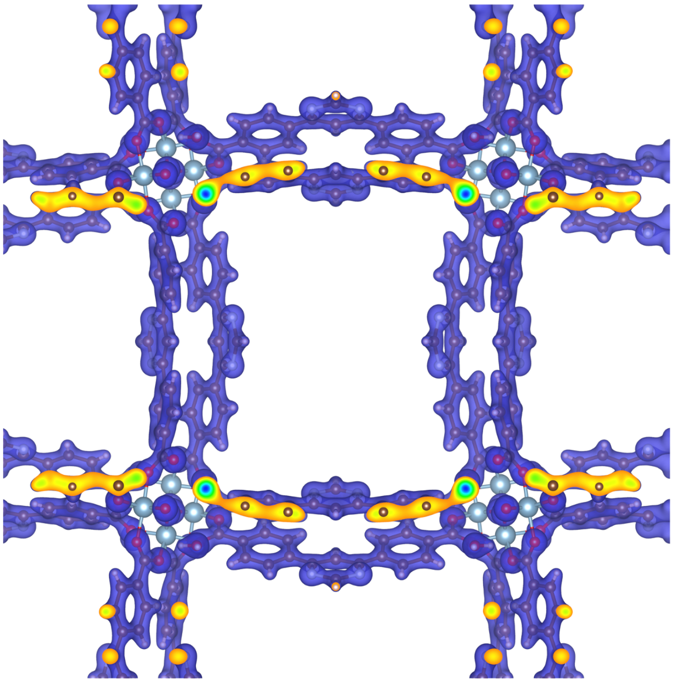

Supplementary Fig. 17 Density-derived electrostatic and chemical (DDEC) charges of ZJU-520 (Al).

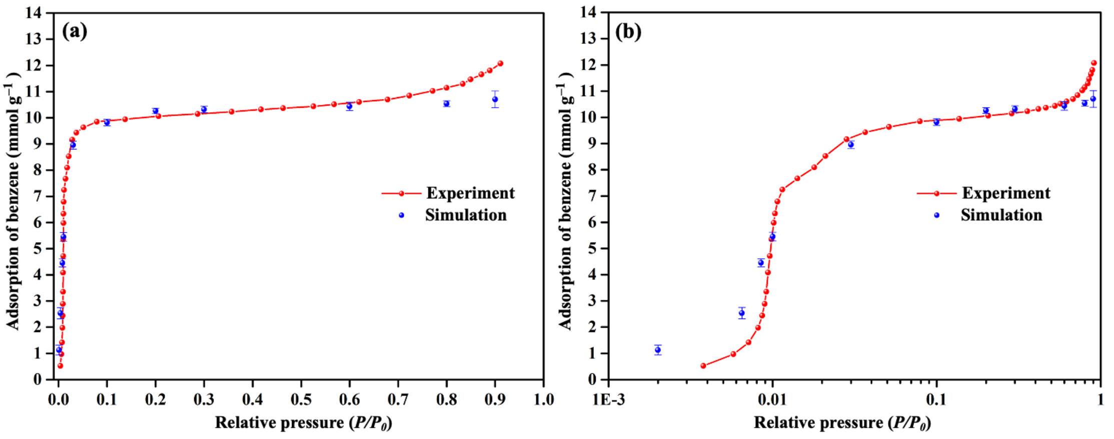

Supplementary Fig. 18 Experimental benzene adsorption isotherm (in red line) and simulated benzene adsorption (in blue dots) at 298 K by ZJU-520(Al) at normalized scale (a) and logarithmic-scale (b).

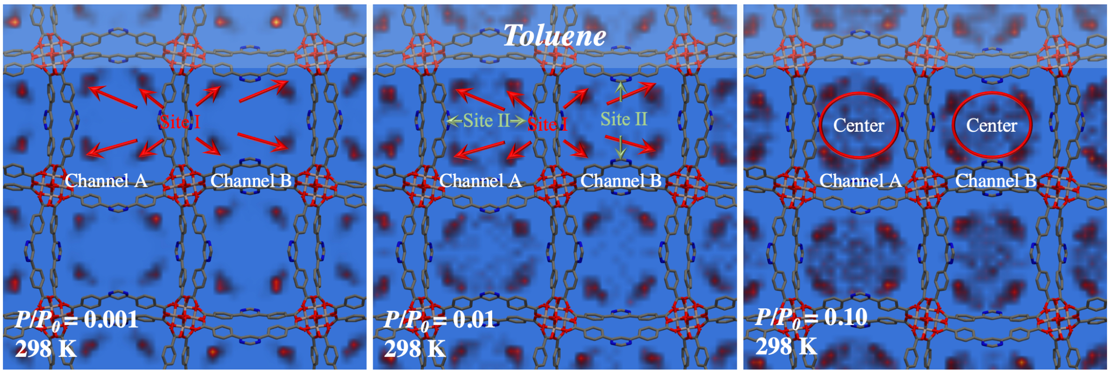

Supplementary Fig. 19 Adsorption process of toluene by ZJU-520(Al) at 298 K using GCMC simulation, as viewed from c axis.

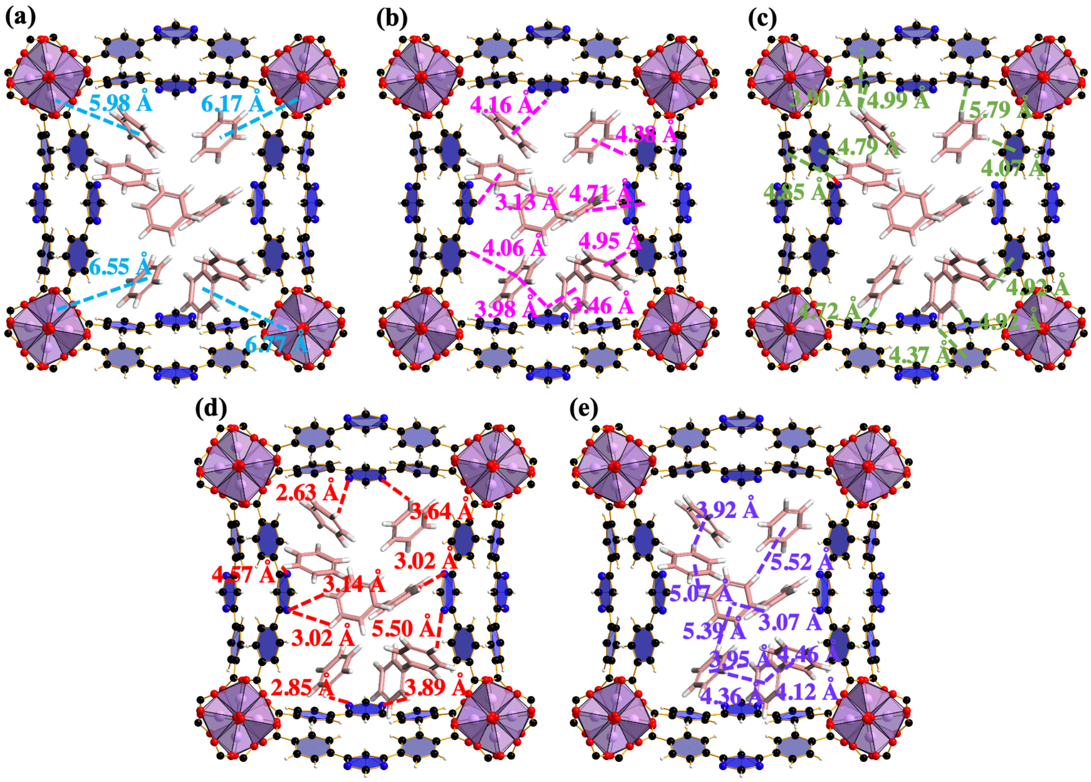

Supplementary Fig. 20 Host – guest interactions, including Al – π interactions (a) (in blue lines), C – H(L) ··· π(Bz) interactions (b) (in pink lines), C – H(Bz) ··· π(L) interactions (c) (in green lines) and C – H(Bz) ··· N(L) interactions (d) (in red lines). Guest – guest interactions through C – H(Bz) ··· π(Bz) interactions (e) (in purple lines) at 298 K and $P/P_0 = 0.01$, as viewed from c axis.

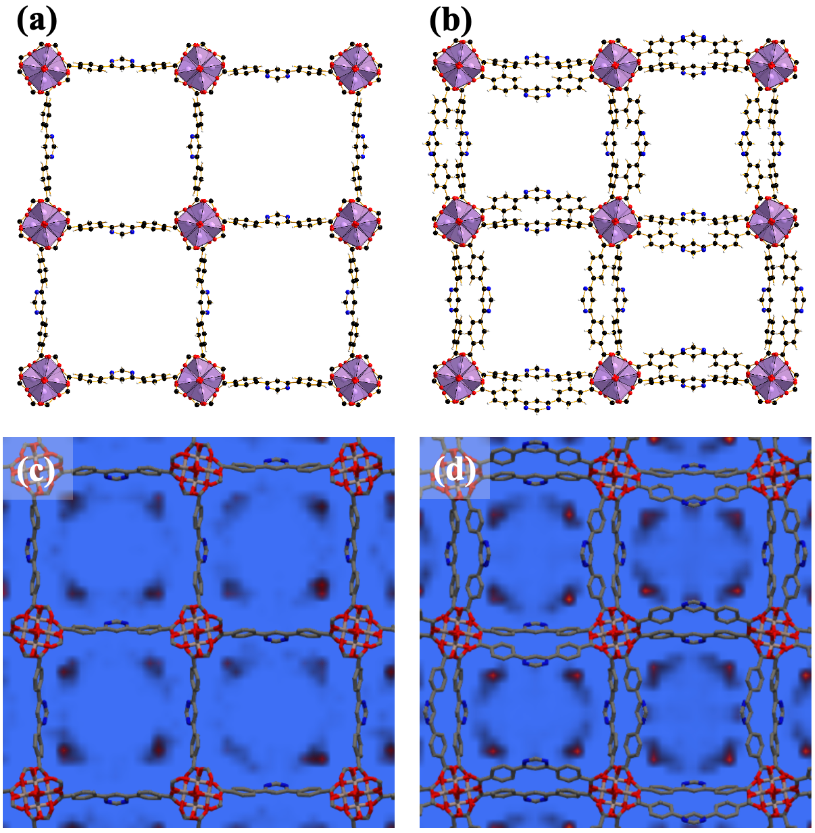

Supplementary Fig. 21 Structure of single-walled (a) and double-walled (b) ZJU-520(Al), and the potential fields of benzene by single-walled (c) and double-walled (d) ZJU-520(Al) at $P/P_0 = 0.01$ and 298 K.

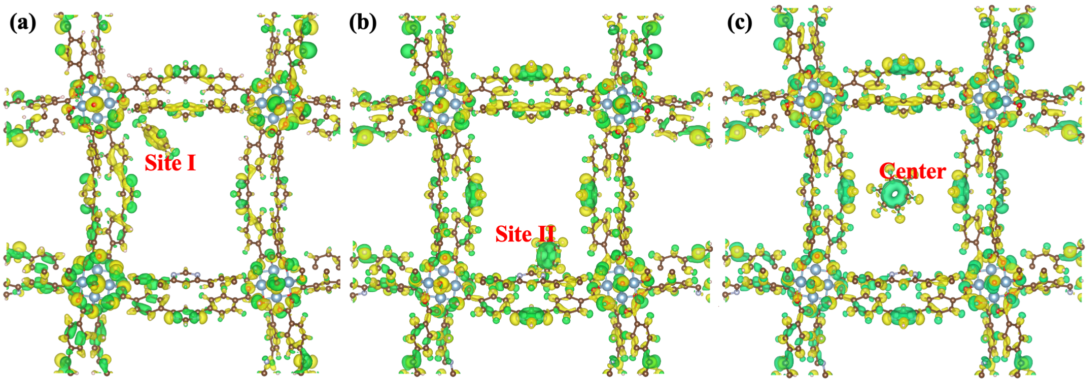

Supplementary Fig. 22 Isosurfaces of charge density difference in ZJU-520(Al) with benzene molecule in site I (a), site II (b) and center (c). Yellow and green demonstrate electronic accumulation and depletion, respectively, as viewed from c axis.

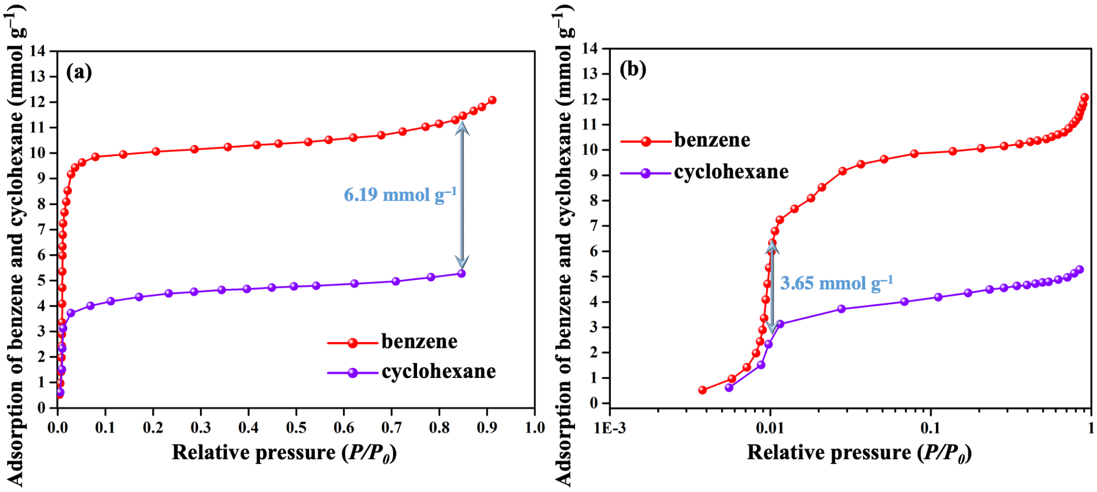

Supplementary Fig. 23 Benzene and cyclohexane adsorption isotherms by ZJU-520(Al) at normalized scale (a) and logarithmic-scale (b).

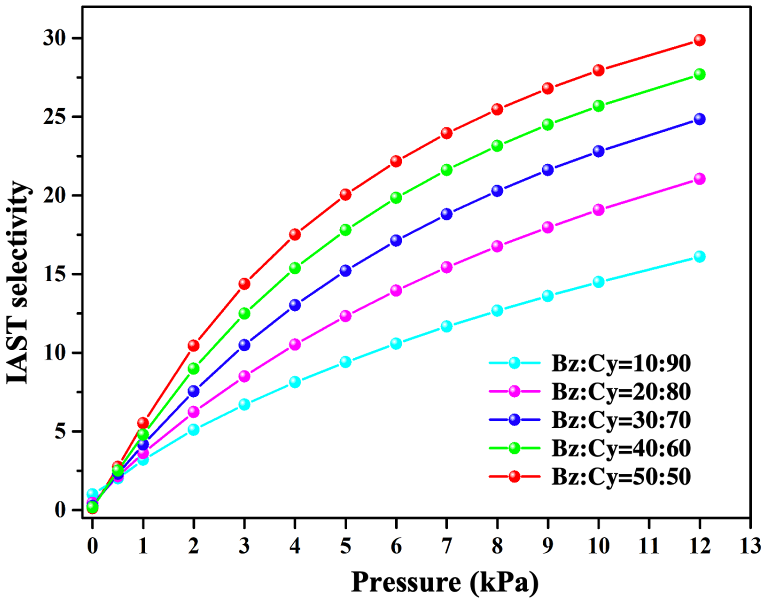

Supplementary Fig. 24 Predicted ideal adsorption solution theory (IAST) selectivity curves of benzene/cyclohexane (Bz/Cy) with Bz:Cy = 10:90, 20:80, 30:70, 40:60, 50:50, respectively.

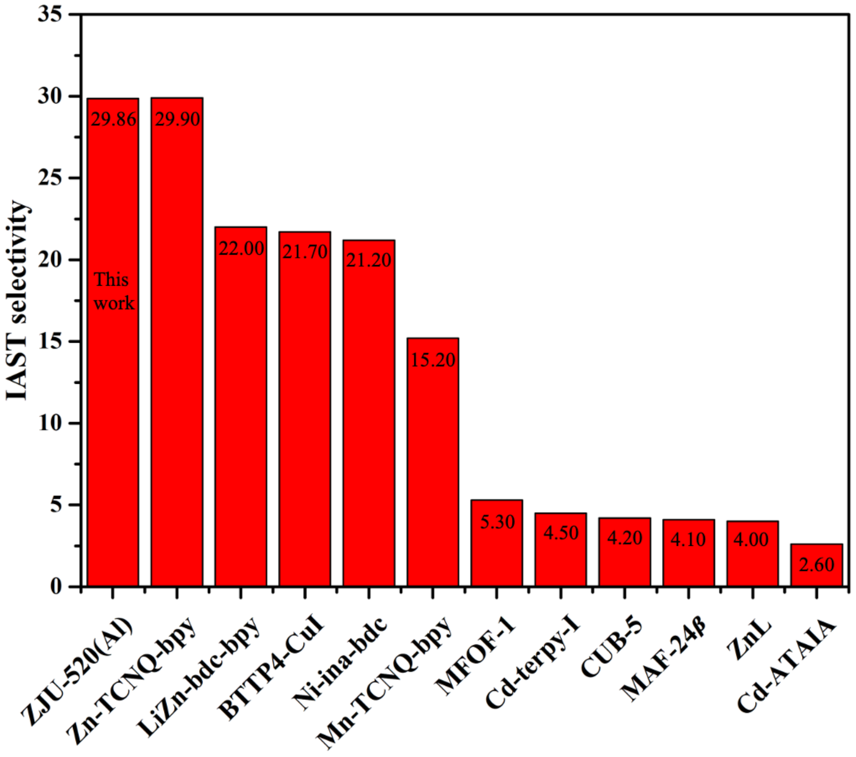

Supplementary Fig. 25 IAST selectivity of benzene/cyclohexane at vapor volume of 50/50 by ZJU-520(Al) and other reported materials [48].

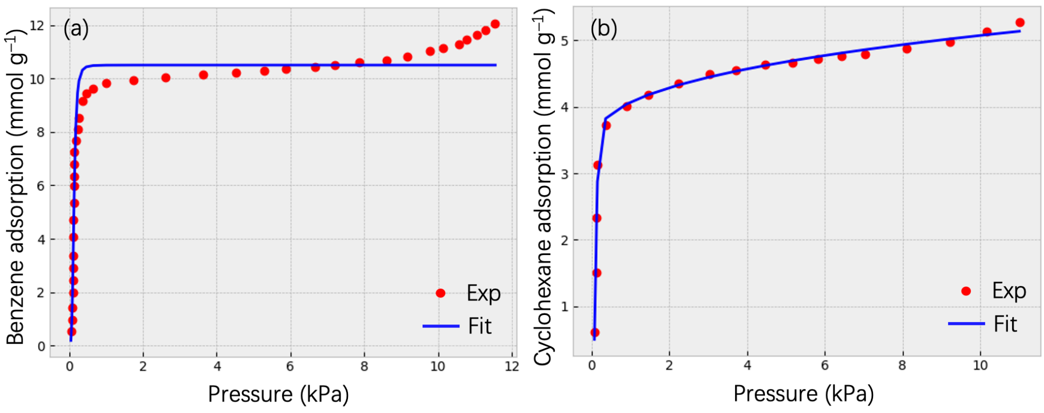

Supplementary Fig 26. Isotherms of benzene (a) and cyclohexane (b) by ZJU-520(Al) at 298 K, respectively. The blue solid lines are fitted by DSLF model.

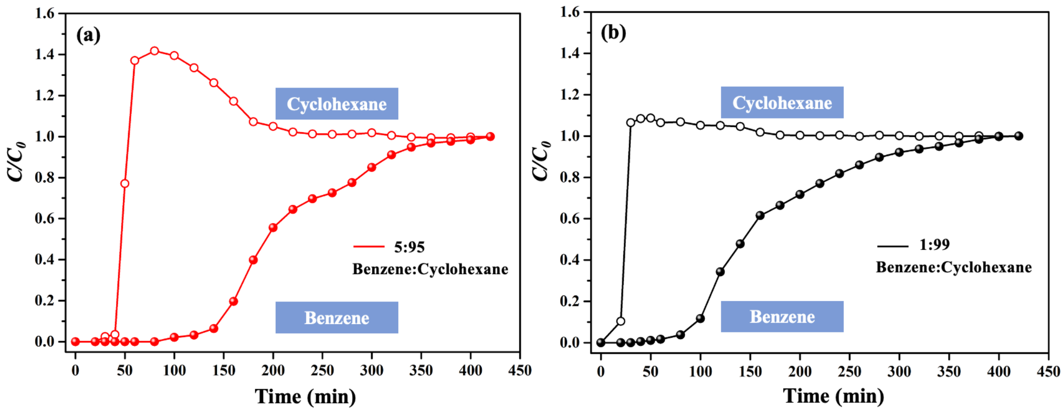

Supplementary Fig 27. Breakthrough curves for benzene/cyclohexane (Bz/Cy) separations, including 5:95 (v/v) (a) and 1:99 (v/v) (b) Bz/Cy mixture separation.

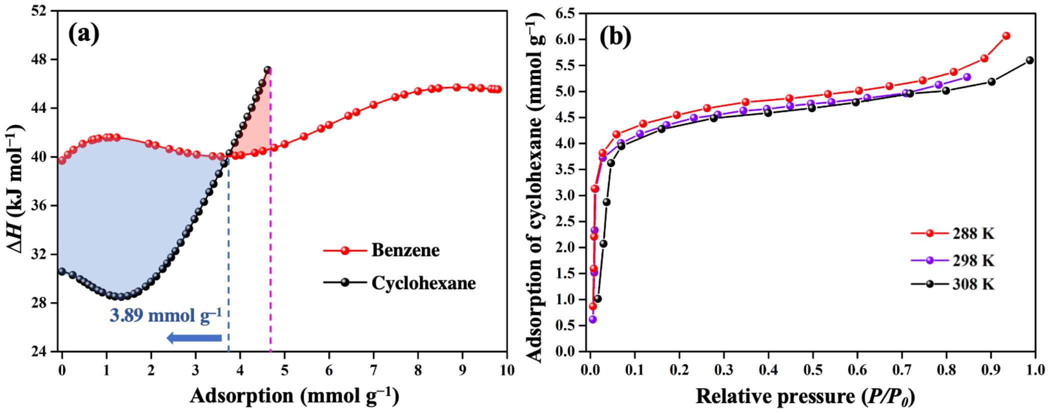

Supplementary Fig. 28 Isosteric heat (ΔH) of cyclohexane (in black line) and benzene (in red line) (a), and adsorption isotherms of cyclohexane at 288, 298 and 308 K (b) by ZJU-520(Al).

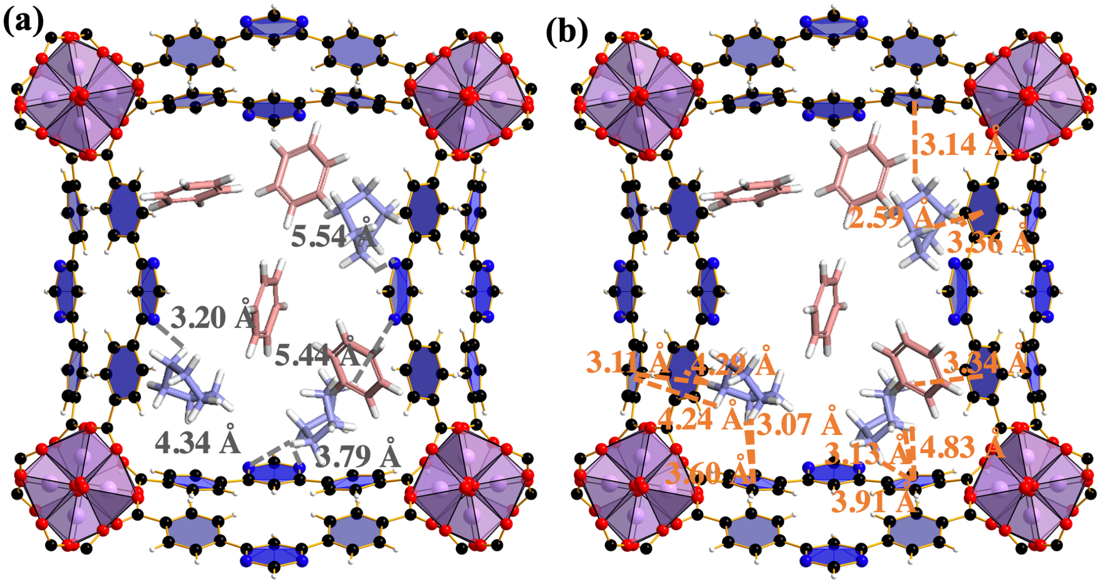

Supplementary Fig. 29 Interactions between framework and cyclohexane, including C – H(Cy) ··· N(L) interactions (a) (in black lines) and C – H(Cy) ··· π(L) interactions (b) (in orange lines), as viewed from c axis.

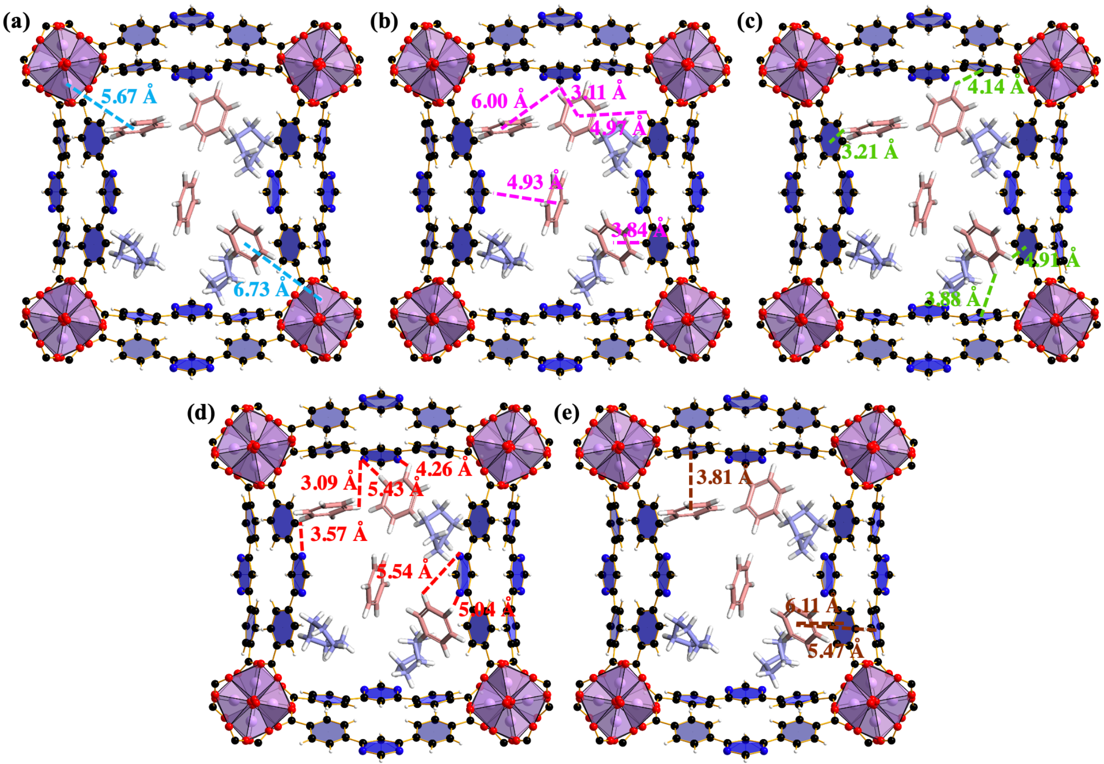

Supplementary Fig. 30 Interactions between framework and benzene, including Al – π interactions (a) (in blue lines), C – H(L) ··· π(Bz) interactions (b) (in pink lines), C – H(Bz) ··· π(L) interactions (c) (in green lines), C – H(Bz) ··· N(L) (d) (in red lines) and π(Bz) ··· π(L) interactions (e) (in brown lines), as viewed from c axis.

Supplementary Table 1. Crystal data and structure refinement for ZJU-520(Al) with and without guest molecules.

Identification code	As-synthesized ZJU-520(Al) with guest molecules	Activated ZJU-520(Al) without guest molecules
Empirical formula	Al(OH)(DBP)·DMF·H_2O ($C_{21}H_{19}AlN_3O_7$)	Al(OH)(DBP) ($C_{18}H_{10}AlN_2O_5$)
Formula weight	452.37	361.37
Temperature/K	100.15	100.15
Crystal system	tetragonal	tetragonal
Space group	$I4_1md$	$I4_1md$
a/Å	36.647(5)	36.647(5)
b/Å	36.647(5)	36.647(5)
c/Å	10.556(2)	10.556(2)
α/°	90	90
β/°	90	90
γ/°	90	90
Volume/Å^3	14177(5)	14177(5)
Z	16	16
ρ_{calc} g/cm^3	0.848	0.677
μ/mm^{-1}	0.764	0.645
F(000)	3760.0	2960.0
Crystal size/mm^3	0.10 × 0.02 × 0.02	0.10 × 0.02 × 0.02
Radiation	CuKα (λ = 1.54178)	CuKα (λ = 1.54178)
2$\boldsymbol{\theta}$ range for data collection/°	6.822 to 107.72	6.822 to 107.72
Index ranges	$-33 \leq h \leq 38$, $-38 \leq k \leq 36$, $-11 \leq l \leq 9$	$-33 \leq h \leq 38$, $-38 \leq k \leq 36$, $-11 \leq l \leq 9$
Reflections collected	34555	34555
Independent reflections	4161 [R_{int} = 0.2076, R_{sigma} = 0.1616]	4161 [R_{int} = 0.2076, R_{sigma} = 0.1616]
Data/restraints/parameters	4161/13/316	4161/26/257
Goodness-of-fit on F^2	1.006	0.987
Final R indexes [I>=2σ (I)]	R_1 = 0.0980, wR_2 = 0.2524	R_1 = 0.0804, wR_2 = 0.2020
Final R indexes [all data]	R_1 = 0.1204, wR_2 = 0.2831	R_1 = 0.1204, wR_2 = 0.2272
Largest diff. peak/hole / e Å^{-3}	0.47/-0.46	0.24/-0.38
Flack parameter	0.56(18)	0.36(15)

$^aR_1=\sum(||F_0|-|F_C||)/\sum|F_0|$;

$^awR_2=[[\sum w(|F_0|^2-|F_C|^2)^2/\sum w(F_0^2)^2]]^{1/2}$, $[F_0>4\boldsymbol{\sigma}(F_0)]$.

Supplementary Table 2. Selected bond length (Å) of ZJU-520(Al).

Atom	Length (Å)	Atom	Length (Å)	Atom	Length (Å)
Al01-O002	1.828(8)	C00G-C00M	1.433(18)	C1-C00Q	1.378(18)
Al01-O0021	1.836(9)	C4-C00U	1.384(18)	C00C-C2	1.405(17)
Al01-O003	1.877(8)	C4-C00U3	1.384(18)	C00C-C00K	1.462(17)
Al01-O004	1.901(8)	C00H-C00I	1.413(16)	C2-C00I	1.365(16)
Al01-O005	1.935(8)	C00H-C00Q	1.369(17)	C00E-C00H	1.475(16)
Al01-O006	1.945(8)	C00J-C00N	1.370(18)	C00G-C5	1.451(15)
N1-C00K	1.424(17)	C00K-C00P	1.384(16)	C00G-C00J	1.418(17)
N1-C00O	1.331(14)	C00M-C00T	1.391(16)	C00U-N9	1.44(3)
O003-C00E	1.282(14)	C00N-C00R	1.401(19)	N9-C10	1.32(4)
O004-C5	1.307(13)	N9A-C10A	1.33(3)	O009-C00L	1.26(2)
O005-C51	1.209(13)	N9A-C00U	1.50(3)	N8-C6	1.53(3)
O006-C00E2	1.266(14)	C00R-C00T	1.394(17)	N8-C7	1.49(3)
C1-C00C	1.389(19)	C00R-C00U	1.500(19)	N8-C00L	1.30(2)

Supplementary Table 3. Selected bond angle (Å) of ZJU-520(Al).

Atom	Angle (˚)	Atom	Angle (˚)	Atom	Angle (˚)
O002-Al01-O0021	92.8(3)	C1-C00C-C2	117.5(11)	C00T-C00M-C00G	119.7(11)
O0021-Al01-O003	93.4(4)	C1-C00C-C00K	121.0(11)	C00J-C00N-C00R	118.0(11)
O002-Al01-O003	173.8(4)	C2-C00C-C00K	121.2(12)	C10A-N9A-C00U	113(3)
O002-Al01-O004	89.3(4)	C00I-C2-C00C	121.4(12)	N13-C00O-N1	127.1(19)
O0021-Al01-O004	177.2(4)	O003-C00E-C00H	119.5(10)	N9A3-C10A-N9A	130(4)
O002-Al01-O005	91.0(4)	O0061-C00E-O003	123.5(10)	C00K3-C00P-C00K	120.7(17)
O0021-Al01-O005	91.6(3)	O0061-C00E-C00H	117.0(11)	C00H-C00Q-C1	120.0(12)
O0021-Al01-O006	89.2(3)	C00J-C00G-C5	124.5(11)	C00N-C00R-C00U	120.4(12)
O002-Al01-O006	93.6(3)	C00J-C00G-C00M	116.1(10)	C00T-C00R-C00N	120.3(11)
O003-Al01-O004	84.5(3)	C00M-C00G-C5	119.4(10)	C00T-C00R-C00U	119.3(12)
O003-Al01-O005	89.6(3)	C00U-C4-C00U3	119.1(18)	C00M-C00T-C00R	121.4(12)
O003-Al01-O006	85.7(3)	C00I-C00H-C00E	119.2(10)	C4-C00U-N9A	117.7(15)
O004-Al01-O005	90.3(3)	C00Q-C00H-C00E	121.0(11)	C4-C00U-C00R	123.1(14)
O004-Al01-O006	88.7(3)	C00Q-C00H-C00I	119.6(11)	C4-C00U-N9	117.8(15)
O005-Al01-O006	175.2(4)	O004-C5-C00G	113.5(10)	C00R-C00U-N9A	112.4(15)
C00O-N1-C00K	116.9(12)	O0052-C5-O004	124.6(9)	N9-C00U-C00R	115.0(14)
Al01-O002-Al012	127.1(4)	O0052-C5-C00G	121.7(10)	C10-N9-C00U	110(3)
C00E-O003-Al01	131.3(7)	C2-C00I-C00H	119.5(11)	N93-C10-N9	129(7)
C5-O004-Al01	130.0(6)	C00N-C00J-C00G	124.4(12)	C7-N8-C6	116(2)
C51-O005-Al01	134.3(7)	N1-C00K-C00C	116.5(10)	C00L-N8-C6	124(2)
C00E2-O006-Al01	133.4(7)	C00P-C00K-N1	119.2(12)	C00L-N8-C7	118(2)
C00Q-C1-C00C	121.7(11)	C00P-C00K-C00C	123.8(13)	O009-C00L-N8	122.3(16)

Supplementary Table 4. The elemental analysis of ZJU-520(Al).

Al	C	N	O	H
9.60 %	52.12 %	5.30 %	26.92 %	6.06 %

Supplementary Table 5. Benzene adsorption by reported adsorbents at 298 K and P/P_0 = 0.01.

Adsorbents	$Q_{0.01}$ (mmol g^{-1})	S_{BET} (m^2 g^{-1})	Reference
**ZJU-520(Al)**	**5.98**	**2235**	**This work**
Double-walled BUT-53(Co)	2.49	811	38
Double-walled BUT-54(Co)	4.31	1128	38
Double-walled BUT-55(Co)	3.51	873	38
Double-walled BUT-56(Co)	3.42	897	38
Double-walled BUT-57(Co)	3.44	970	38
Double-walled BUT-58(Zn)	3.41	849	38
ZJU-620(Al)	3.80	1324	19
Mn-MOF-74	6.30	1500	31
[Li_2Zn_2(1-H-bdc)$_3$(bpy)]	2.44	1052	49
[Li_2Zn_2(1-NH_2-bdc)$_3$(bpy)]	2.05	876	49
[Li_2Zn_2(1-NO_2-bdc)$_3$(bpy)]	1.22	742	49
MIL-101(Cr)	1.09	3980	50
Carboxen 1000	2.81	1200	50
ZIF-8(Zn)	0.01	1345	50
MCM-41	0.42	1046	50
PAF-1	3.66	4201	50
BUT-66(Zr)	2.72	1096	50
BUT-67(Zr)	1.95	984	50
MOF-177(Zn)	1.60	2970	51
HKUST-1	0.91	1707	52
SBA-15	0.63	805	53
HZSM-5	1.35	550	53
Active carbon	2.35	1600	53
IISERP-COF2	0.13	584	54
IISERP-COF4	0.063	337	54
UiO-66-(Cu^{II})	3.92	1240	55
MFM-300(Sc)	3.02	1228	55
MFM-300(VIII)	2.28	1461	55
MFM-300(Cr)	2.71	1415	55
MFM-300(Fe)	2.63	1062	55
MFM-300(Al)	2.99	1370	55
MFM-300(Ga)	3.01	1242	55
MFM-300(In)	2.82	1084	55
UiO-66-(Cu^{I})	2.74	1201	55
UiO-66-defect	2.55	1313	55
UiO-66	1.78	996	55

Supplementary Table 6. Theorical interplanar spacing obtained from the crystal information file of ZJU-520(Al).

**(*h*, *k*, *l*)**	**2θ**	**Interplanar spacing (Å)**	**(*h*, *k*, *l*)**	**2θ**	**Interplanar spacing (Å)**
(2,2,0)	6.82	12.96	(1,5,0)	12.31	7.19
(3,1,0)	7.62	11.59	(1,4,1)	13.01	6.80
(1,0,1)	8.71	10.14	(4,1,1)	13.01	6.80
(4,0,0)	9.65	9.16	(4,4,0)	13.66	6.48
(2,1,1)	9.96	8.87	(3,5,0)	14.08	6.28
(1,2,1)	9.96	8.87	(5,3,0)	14.08	6.28
(4,2,0)	10.79	8.19	(0,6,0)	14.49	6.11
(0,3,1)	11.07	7.99	(3,4,1)	14.70	6.02
(3,0,1)	11.07	7.99	(6,2,0)	15.28	5.79
(2,3,1)	12.08	7.32	(5,2,1)	15.48	5.72
(3,2,1)	12.08	7.32	(6,1,1)	16.93	5.23
(5,1,0)	12.31	7.19	(7,1,0)	17.10	5.18

Supplementary Table 7. Restrained electrostatic potential (RESP) charges of H_2DBP ligand.

**Atom Number**	**Charge (*e*)**	**Atom Number**	**Charge (*e*)**	**Atom Number**	**Charge (*e*)**
C1	0.468	C13	-0.267	H1	0.430
C2	0.217	C14	0.215	H2	0.173
C3	-0.266	C15	-0.244	H3	0.188
C4	-0.045	C16	0.040	H4	0.115
C5	-0.301	C17	-0.303	H5	0.126
C6	0.041	C18	0.469	H6	0.251
C7	-0.255	O1	-0.470	H7	0.046
C8	0.854	O2	-0.545	H8	0.125
C9	-0.864	O3	-0.469	H9	0.173
C10	0.839	O4	-0.546	H10	0.114
C11	0.609	N1	-0.748	H11	0.189
C12	-0.044	N2	-0.745	H12	0.430

Supplementary Table 8. Density-derived electrostatic and chemical (DDEC) charges of ZJU-520(Al).

Atom	x	y	z	Partial charge	Atom	x	y	z	Partial charge	Atom	x	y	z	Partial charge	Atom	x	y	z	Partial charge
Al1	26.11	27.63	2.94	1.59	C13	22.15	27.65	0.50	-0.10	C169	32.77	10.42	10.36	-0.08	H37	14.44	8.72	1.41	0.12
Al2	7.79	9.30	8.22	1.59	C14	20.86	30.02	6.57	0.06	C170	35.45	8.84	3.14	0.23	H38	14.72	5.63	7.99	0.13
Al3	28.86	27.35	8.22	1.59	C15	22.01	30.46	7.23	-0.12	C171	31.69	11.77	1.51	-0.08	H39	7.22	8.48	0.92	0.42
Al4	10.53	9.02	2.94	1.59	C16	19.62	30.41	7.33	0.20	C172	34.02	10.80	0.22	-0.13	H40	7.14	7.58	1.96	0.42
Al5	9.02	7.79	5.58	1.59	C17	2.61	9.28	5.14	-0.12	C173	32.82	9.32	5.78	-0.10	H41	8.84	1.82	2.99	0.11
Al6	27.35	26.11	0.30	1.59	C18	2.51	8.96	3.79	0.05	C174	34.11	11.70	1.29	0.06	H42	9.90	3.65	10.10	0.11
Al7	9.30	10.53	0.30	1.59	C19	3.69	8.64	3.11	-0.11	C175	32.96	12.13	1.95	-0.12	H43	9.90	5.69	0.62	0.11
Al8	27.63	28.86	5.58	1.59	C20	6.25	8.82	5.82	0.62	C176	35.45	12.15	1.79	0.20	H44	8.50	3.94	6.99	0.12
Al9	26.11	9.02	2.94	1.59	C21	5.09	10.86	0.41	-0.03	C177	15.71	27.61	10.42	-0.12	H45	6.21	5.73	9.85	0.13
Al10	7.79	27.35	8.22	1.59	C22	4.97	8.96	5.11	-0.01	C178	15.82	27.29	9.07	0.05	H46	7.85	1.85	7.71	0.11
Al11	28.86	9.30	8.22	1.59	C23	6.41	10.48	10.51	0.62	C179	14.63	26.97	8.38	-0.11	H47	8.72	3.88	4.05	0.12
Al12	10.53	27.63	2.94	1.59	C24	4.90	8.64	3.74	-0.08	C180	12.07	27.14	0.55	0.62	H48	5.63	3.60	0.07	0.12
Al13	9.02	28.86	5.58	1.59	C25	3.88	10.42	10.36	-0.08	C181	13.24	29.18	5.68	-0.03	H49	8.48	11.11	3.56	0.42
Al14	27.35	10.53	0.30	1.59	C26	1.20	8.84	3.14	0.23	C182	13.35	27.28	10.39	-0.01	H50	7.58	11.19	4.59	0.42
Al15	9.30	26.11	0.30	1.59	C27	4.96	11.77	1.51	-0.08	C183	11.91	28.80	5.23	0.62	H51	27.17	20.15	8.27	0.11
Al16	27.63	7.79	5.58	1.59	C28	2.63	10.80	0.22	-0.13	C184	13.42	26.96	9.02	-0.08	H52	28.23	21.97	4.82	0.11
N1	19.52	26.60	7.11	-0.34	C29	3.83	9.32	5.78	-0.10	C185	14.44	28.75	5.09	-0.08	H53	28.23	24.02	5.90	0.11
N2	19.64	30.98	8.72	-0.33	C30	2.54	11.70	1.29	0.06	C186	17.12	27.16	8.42	0.23	H54	26.82	22.26	1.71	0.12
N3	1.19	8.28	1.84	-0.34	C31	3.69	12.13	1.95	-0.12	C187	13.37	30.09	6.78	-0.08	H55	24.53	24.05	4.57	0.13
N4	1.21	12.46	3.26	-0.33	C32	1.20	12.15	1.79	0.20	C188	15.69	29.13	5.50	-0.13	H56	26.18	20.17	2.43	0.11
N5	35.46	28.37	1.84	-0.34	C33	34.03	27.36	5.14	-0.12	C189	14.49	27.65	0.50	-0.10	H57	27.05	22.20	9.33	0.12
N6	35.44	24.19	3.26	-0.33	C34	34.14	27.68	3.79	0.05	C190	15.79	30.02	6.57	0.06	H58	23.95	21.93	5.35	0.13
N7	17.13	10.05	7.11	-0.34	C35	32.96	28.01	3.11	-0.11	C191	14.64	30.46	7.23	-0.12	H59	26.80	29.43	8.84	0.42

N8	17.01	5.67	8.72	-0.33	C36	30.40	27.83	5.82	0.62	C192	17.03	30.40	7.33	0.20	H60	25.90	29.51	9.87	0.42
N9	10.05	1.19	9.75	-0.34	C37	31.56	25.79	0.41	-0.03	C193	9.04	34.03	2.50	-0.12	H61	9.48	16.50	8.27	0.11
N10	5.87	1.21	0.62	-0.33	C38	31.68	27.69	5.11	-0.01	C194	9.36	34.14	1.15	0.05	H62	8.42	14.67	4.82	0.11
N11	28.37	19.52	4.48	-0.34	C39	30.23	26.17	10.51	0.62	C195	9.68	32.96	0.47	-0.11	H63	8.42	12.63	5.90	0.11
N12	23.99	19.64	6.09	-0.33	C40	31.75	28.01	3.74	-0.08	C196	9.51	30.40	3.18	0.62	H64	9.83	14.38	1.71	0.12
N13	8.28	17.13	4.48	-0.34	C41	32.77	26.23	10.36	-0.08	C197	7.47	31.56	8.32	-0.03	H65	12.12	12.60	4.57	0.13
N14	12.65	17.02	6.07	-0.33	C42	35.45	27.81	3.14	0.23	C198	9.37	31.68	2.47	-0.01	H66	10.47	16.47	2.43	0.11
N15	26.60	35.46	9.75	-0.34	C43	31.69	24.88	1.51	-0.08	C199	7.85	30.23	7.87	0.62	H67	9.60	14.44	9.33	0.12
N16	30.78	35.44	0.62	-0.33	C44	34.02	25.84	0.22	-0.13	C200	9.69	31.75	1.10	-0.08	H68	12.69	14.72	5.35	0.13
N17	19.52	10.05	7.11	-0.34	C45	32.82	27.32	5.78	-0.10	C201	7.90	32.77	7.72	-0.08	H69	9.84	7.22	8.84	0.42
N18	19.63	5.67	8.72	-0.33	C46	34.11	24.95	1.29	0.06	C202	9.48	35.45	0.51	0.23	H70	10.75	7.14	9.87	0.42
N19	1.19	28.37	1.84	-0.34	C47	32.96	24.51	1.95	-0.12	C203	6.56	31.69	9.42	-0.08	H71	27.81	34.82	2.99	0.11
N20	1.21	24.19	3.26	-0.33	C48	35.45	24.50	1.79	0.20	C204	7.52	34.02	8.14	-0.13	H72	26.75	33.00	10.10	0.11
N21	35.46	8.28	1.84	-0.34	C49	15.71	9.04	10.42	-0.12	C205	9.00	32.82	3.14	-0.10	H73	26.74	30.96	0.62	0.11
N22	35.44	12.46	3.26	-0.33	C50	15.82	9.36	9.07	0.05	C206	6.63	34.11	9.21	0.06	H74	28.15	32.71	6.99	0.12
N23	17.13	26.60	7.11	-0.34	C51	14.63	9.68	8.38	-0.11	C207	6.19	32.96	9.87	-0.12	H75	30.44	30.92	9.85	0.13
N24	17.01	30.98	8.72	-0.33	C52	12.07	9.51	0.55	0.62	C208	6.18	35.45	9.71	0.20	H76	28.80	34.80	7.71	0.11
N25	10.05	35.46	9.75	-0.34	C53	13.24	7.47	5.68	-0.03	C209	27.36	15.71	7.78	-0.12	H77	27.92	32.77	4.05	0.12
N26	5.87	35.44	0.62	-0.33	C54	13.35	9.37	10.39	-0.01	C210	27.68	15.82	6.43	0.05	H78	31.02	33.04	0.07	0.12
N27	28.37	17.13	4.48	-0.34	C55	11.91	7.85	5.23	0.62	C211	28.01	14.63	5.75	-0.11	H79	28.17	25.54	3.56	0.42
N28	23.99	17.01	6.09	-0.33	C56	13.42	9.69	9.02	-0.08	C212	27.83	12.07	8.46	0.62	H80	29.07	25.46	4.59	0.42
N29	8.28	19.52	4.48	-0.34	C57	14.44	7.90	5.09	-0.08	C213	25.79	13.24	3.05	-0.03	H81	20.15	8.84	0.35	0.11
N30	12.65	19.63	6.07	-0.33	C58	17.12	9.48	8.42	0.23	C214	27.69	13.35	7.75	-0.01	H82	21.97	9.90	7.46	0.11
N31	26.60	1.19	9.75	-0.34	C59	13.37	6.56	6.78	-0.08	C215	26.17	11.91	2.59	0.62	H83	24.02	9.90	8.54	0.11
N32	30.78	1.21	0.62	-0.33	C60	15.69	7.52	5.50	-0.13	C216	28.01	13.42	6.38	-0.08	H84	22.26	8.50	4.35	0.12

O1	27.30	27.55	4.33	-0.77	C61	14.49	9.00	0.50	-0.10	C217	26.23	14.44	2.45	-0.08	H85	24.05	6.21	7.21	0.13
O2	24.76	27.76	1.65	-0.58	C62	15.79	6.63	6.57	0.06	C218	27.81	17.12	5.78	0.23	H86	20.17	7.85	5.07	0.11
O3	24.73	28.03	4.18	-0.58	C63	14.64	6.19	7.23	-0.12	C219	24.88	13.37	4.15	-0.08	H87	22.20	8.72	1.41	0.12
O4	25.74	25.74	3.12	-0.52	C64	17.03	6.23	7.32	0.19	C220	25.84	15.69	2.86	-0.13	H88	21.93	5.63	7.99	0.13
O5	26.37	29.53	2.67	-0.56	C65	9.04	2.61	2.50	-0.12	C221	27.32	14.49	8.42	-0.10	H89	29.43	8.48	0.92	0.42
O6	29.43	29.02	1.09	-0.78	C66	9.36	2.51	1.15	0.05	C222	24.95	15.79	3.93	0.06	H90	29.51	7.58	1.96	0.42
O7	8.98	9.23	9.61	-0.77	C67	9.68	3.69	0.47	-0.11	C223	24.51	14.64	4.59	-0.12	H91	1.82	27.17	5.63	0.11
O8	6.43	9.44	6.93	-0.58	C68	9.51	6.25	3.18	0.62	C224	24.57	17.03	4.69	0.20	H92	3.65	28.23	2.18	0.11
O9	6.40	9.70	9.46	-0.58	C69	7.47	5.09	8.32	-0.03	C225	9.28	20.94	7.78	-0.12	H93	5.69	28.23	3.26	0.11
O10	7.41	7.41	8.40	-0.52	C70	9.37	4.97	2.47	-0.01	C226	8.96	20.83	6.43	0.05	H94	3.94	26.82	9.63	0.12
O11	8.04	11.21	7.95	-0.56	C71	7.85	6.41	7.87	0.62	C227	8.64	22.01	5.75	-0.11	H95	5.73	24.53	1.93	0.13
O12	11.11	10.70	6.37	-0.78	C72	9.69	4.90	1.10	-0.08	C228	8.82	24.58	8.46	0.62	H96	1.85	26.18	10.34	0.11
O13	27.67	27.42	9.61	-0.77	C73	7.90	3.88	7.72	-0.08	C229	10.86	23.41	3.05	-0.03	H97	3.88	27.05	6.69	0.12
O14	30.21	27.21	6.93	-0.58	C74	9.48	1.20	0.51	0.23	C230	8.96	23.29	7.75	-0.01	H98	3.60	23.95	2.71	0.12
O15	30.24	26.94	9.46	-0.58	C75	6.56	4.96	9.42	-0.08	C231	10.48	24.74	2.59	0.62	H99	11.11	26.80	6.20	0.42
O16	29.23	29.24	8.40	-0.52	C76	7.52	2.63	8.14	-0.13	C232	8.64	23.22	6.38	-0.08	H100	11.19	25.90	7.23	0.42
O17	28.61	25.44	7.95	-0.56	C77	9.00	3.83	3.14	-0.10	C233	10.42	22.20	2.45	-0.08	H101	34.82	9.48	5.63	0.11
O18	25.54	25.95	6.37	-0.78	C78	6.63	2.54	9.21	0.06	C234	8.84	19.53	5.78	0.23	H102	33.00	8.42	2.18	0.11
O19	9.35	9.10	4.33	-0.77	C79	6.19	3.69	9.87	-0.12	C235	11.77	23.28	4.15	-0.08	H103	30.96	8.42	3.26	0.11
O20	11.89	8.89	1.65	-0.58	C80	6.18	1.20	9.71	0.20	C236	10.80	20.96	2.86	-0.13	H104	32.71	9.83	9.63	0.12
O21	11.92	8.62	4.18	-0.58	C81	27.36	20.94	7.78	-0.12	C237	9.32	22.15	8.42	-0.10	H105	30.92	12.12	1.93	0.13
O22	10.91	10.91	3.12	-0.52	C82	27.68	20.83	6.43	0.05	C238	11.70	20.86	3.93	0.06	H106	34.80	10.47	10.34	0.11
O23	10.28	7.11	2.67	-0.56	C83	28.01	22.01	5.75	-0.11	C239	12.13	22.01	4.59	-0.12	H107	32.77	9.60	6.69	0.12
O24	7.22	7.63	1.09	-0.78	C84	27.83	24.58	8.46	0.62	C240	12.11	19.61	4.66	0.20	H108	33.04	12.69	2.71	0.12
O25	9.10	8.98	6.97	-0.77	C85	25.79	23.41	3.05	-0.03	C241	27.61	2.61	2.50	-0.12	H109	25.54	9.84	6.20	0.42

O26	8.89	6.43	4.29	-0.58	C86	27.69	23.29	7.75	-0.01	C242	27.29	2.51	1.15	0.05	H110	25.46	10.75	7.23	0.42
O27	8.62	6.40	6.82	-0.58	C87	26.17	24.74	2.59	0.62	C243	26.97	3.69	0.47	-0.11	H111	16.50	27.81	0.35	0.11
O28	10.91	7.41	5.76	-0.52	C88	28.01	23.22	6.38	-0.08	C244	27.14	6.25	3.18	0.62	H112	14.67	26.75	7.46	0.11
O29	7.11	8.04	5.31	-0.56	C89	26.23	22.20	2.45	-0.08	C245	29.18	5.09	8.32	-0.03	H113	12.63	26.74	8.54	0.11
O30	7.63	11.11	3.73	-0.78	C90	27.81	19.53	5.78	0.23	C246	27.28	4.97	2.47	-0.01	H114	14.38	28.15	4.35	0.12
O31	27.42	27.30	1.69	-0.77	C91	24.88	23.28	4.15	-0.08	C247	28.80	6.41	7.87	0.62	H115	12.60	30.44	7.21	0.13
O32	27.21	24.76	9.57	-0.58	C92	25.84	20.96	2.86	-0.13	C248	26.96	4.90	1.10	-0.08	H116	16.47	28.80	5.07	0.11
O33	26.94	24.73	1.54	-0.58	C93	27.32	22.15	8.42	-0.10	C249	28.75	3.88	7.72	-0.08	H117	14.44	27.92	1.41	0.12
O34	29.24	25.74	0.48	-0.52	C94	24.95	20.86	3.93	0.06	C250	27.16	1.20	0.51	0.23	H118	14.72	31.02	7.99	0.13
O35	25.44	26.37	0.03	-0.56	C95	24.51	22.01	4.59	-0.12	C251	30.09	4.96	9.42	-0.08	H119	7.22	28.17	0.92	0.42
O36	25.95	29.43	9.01	-0.78	C96	24.57	19.62	4.69	0.20	C252	29.13	2.63	8.14	-0.13	H120	7.14	29.07	1.96	0.42
O37	9.23	9.35	1.69	-0.77	C97	9.28	15.71	7.78	-0.12	C253	27.65	3.83	3.14	-0.10	H121	8.84	34.82	2.99	0.11
O38	9.44	11.89	9.57	-0.58	C98	8.96	15.82	6.43	0.05	C254	30.02	2.54	9.21	0.06	H122	9.90	33.00	10.10	0.11
O39	9.70	11.92	1.54	-0.58	C99	8.64	14.63	5.75	-0.11	C255	30.46	3.69	9.87	-0.12	H123	9.90	30.96	0.62	0.11
O40	7.41	10.91	0.48	-0.52	C100	8.82	12.07	8.46	0.62	C256	30.47	1.20	9.71	0.20	H124	8.50	32.71	6.99	0.12
O41	11.21	10.28	0.03	-0.56	C101	10.86	13.24	3.05	-0.03	C257	18.32	30.15	6.61	-0.25	H125	6.21	30.92	9.85	0.13
O42	10.70	7.22	9.01	-0.78	C102	8.96	13.35	7.75	-0.01	C258	18.32	26.36	6.57	0.22	H126	7.85	34.80	7.71	0.11
O43	27.55	27.67	6.97	-0.77	C103	10.48	11.91	2.59	0.62	C259	18.32	31.27	9.42	0.15	H127	8.72	32.77	4.05	0.12
O44	27.76	30.21	4.29	-0.58	C104	8.64	13.42	6.38	-0.08	C260	18.32	27.41	9.06	-0.27	H128	5.63	33.04	0.07	0.12
O45	28.03	30.24	6.82	-0.58	C105	10.42	14.44	2.45	-0.08	C261	0.00	24.93	1.24	-0.25	H129	8.48	25.54	3.56	0.42
O46	25.74	29.23	5.76	-0.52	C106	8.84	17.12	5.78	0.23	C262	0.00	28.61	1.30	0.21	H130	7.58	25.46	4.59	0.42
O47	29.53	28.61	5.31	-0.56	C107	11.77	13.37	4.15	-0.08	C263	0.00	23.87	3.73	0.21	H131	27.17	16.50	8.27	0.11
O48	29.02	25.54	3.73	-0.78	C108	10.80	15.69	2.86	-0.13	C264	0.00	27.57	3.78	-0.26	H132	28.23	14.67	4.82	0.11
O49	27.30	9.10	4.33	-0.77	C109	9.32	14.49	8.42	-0.10	C265	6.60	0.00	9.15	-0.25	H133	28.23	12.63	5.90	0.11
O50	24.76	8.89	1.65	-0.58	C110	11.70	15.79	3.93	0.06	C266	10.29	0.00	9.21	0.21	H134	26.82	14.38	1.71	0.12

O51	24.73	8.62	4.18	-0.58	C111	12.13	14.64	4.59	-0.12	C267	5.55	0.00	1.09	0.21	H135	24.53	12.60	4.57	0.13
O52	25.74	10.91	3.12	-0.52	C112	12.11	17.04	4.66	0.20	C268	9.24	0.00	1.15	-0.26	H136	26.18	16.47	2.43	0.11
O53	26.37	7.11	2.67	-0.56	C113	27.61	34.03	2.50	-0.12	C269	24.82	18.32	3.97	-0.25	H137	27.05	14.44	9.33	0.12
O54	29.43	7.63	1.09	-0.78	C114	27.29	34.14	1.15	0.05	C270	28.61	18.32	3.94	0.22	H138	23.95	14.72	5.35	0.13
O55	8.98	27.42	9.61	-0.77	C115	26.97	32.96	0.47	-0.11	C271	23.70	18.32	6.78	0.15	H139	26.80	7.22	8.84	0.42
O56	6.43	27.21	6.93	-0.58	C116	27.14	30.40	3.18	0.62	C272	27.57	18.32	6.42	-0.27	H140	25.90	7.14	9.87	0.42
O57	6.40	26.94	9.46	-0.58	C117	29.18	31.56	8.32	-0.03	C273	11.91	18.32	3.92	-0.25	H141	9.48	20.15	8.27	0.11
O58	7.41	29.24	8.40	-0.52	C118	27.28	31.68	2.47	-0.01	C274	8.03	18.32	3.94	0.22	H142	8.42	21.97	4.82	0.11
O59	8.04	25.44	7.95	-0.56	C119	28.80	30.23	7.87	0.62	C275	12.93	18.33	6.77	0.15	H143	8.42	24.02	5.90	0.11
O60	11.11	25.95	6.37	-0.78	C120	26.96	31.75	1.10	-0.08	C276	9.08	18.32	6.42	-0.27	H144	9.83	22.26	1.71	0.12
O61	27.67	9.23	9.61	-0.77	C121	28.75	32.77	7.72	-0.08	C277	30.04	0.00	9.15	-0.25	H145	12.12	24.05	4.57	0.13
O62	30.21	9.44	6.93	-0.58	C122	27.16	35.45	0.51	0.23	C278	26.36	0.00	9.21	0.21	H146	10.47	20.17	2.43	0.11
O63	30.24	9.70	9.46	-0.58	C123	30.09	31.69	9.42	-0.08	C279	31.10	0.00	1.09	0.21	H147	9.60	22.20	9.33	0.12
O64	29.23	7.41	8.40	-0.52	C124	29.13	34.02	8.14	-0.13	C280	27.41	0.00	1.15	-0.26	H148	12.69	21.93	5.35	0.12
O65	28.61	11.21	7.95	-0.56	C125	27.65	32.82	3.14	-0.10	C281	18.32	6.47	6.59	-0.25	H149	9.84	29.43	8.84	0.42
O66	25.54	10.70	6.37	-0.78	C126	30.02	34.11	9.21	0.06	C282	18.32	10.29	6.57	0.22	H150	10.75	29.51	9.87	0.42
O67	9.35	27.55	4.33	-0.77	C127	30.46	32.96	9.87	-0.12	C283	18.32	5.38	9.42	0.15	H151	27.81	1.82	2.99	0.11
O68	11.89	27.76	1.65	-0.58	C128	30.47	35.45	9.71	0.20	C284	18.32	9.24	9.06	-0.27	H152	26.75	3.65	10.10	0.11
O69	11.92	28.03	4.18	-0.58	C129	20.94	9.04	10.42	-0.12	C285	0.00	11.72	1.24	-0.25	H153	26.74	5.69	0.62	0.11
O70	10.91	25.74	3.12	-0.52	C130	20.83	9.36	9.07	0.05	C286	0.00	8.03	1.30	0.21	H154	28.15	3.94	6.99	0.12
O71	10.28	29.53	2.67	-0.56	C131	22.01	9.68	8.38	-0.11	C287	0.00	12.78	3.73	0.21	H155	30.44	5.73	9.85	0.13
O72	7.22	29.02	1.09	-0.78	C132	24.58	9.51	0.55	0.62	C288	0.00	9.08	3.78	-0.26	H156	28.80	1.85	7.71	0.11
O73	9.10	27.67	6.97	-0.77	C133	23.41	7.47	5.68	-0.03	H1	20.15	27.81	0.35	0.11	H157	27.92	3.88	4.05	0.12
O74	8.89	30.21	4.29	-0.58	C134	23.29	9.37	10.39	-0.01	H2	21.97	26.75	7.46	0.11	H158	31.02	3.60	0.07	0.12
O75	8.62	30.24	6.82	-0.58	C135	24.74	7.85	5.23	0.62	H3	24.02	26.74	8.54	0.11	H159	28.17	11.11	3.56	0.42

O76	10.91	29.23	5.76	-0.52	C136	23.22	9.69	9.02	-0.08	H4	22.26	28.15	4.35	0.12	H160	29.07	11.19	4.59	0.42
O77	7.11	28.61	5.31	-0.56	C137	22.20	7.90	5.09	-0.08	H5	24.05	30.44	7.21	0.13	H161	18.32	29.73	5.55	0.14
O78	7.63	25.54	3.73	-0.78	C138	19.53	9.48	8.42	0.23	H6	20.17	28.80	5.07	0.11	H162	18.32	25.96	5.71	0.08
O79	27.42	9.35	1.69	-0.77	C139	23.28	6.56	6.78	-0.08	H7	22.20	27.92	1.41	0.12	H163	18.32	31.71	10.48	0.14
O80	27.21	11.89	9.57	-0.58	C140	20.96	7.52	5.50	-0.13	H8	21.93	31.02	7.99	0.13	H164	18.32	27.74	9.95	0.13
O81	26.94	11.92	1.54	-0.58	C141	22.15	9.00	0.50	-0.10	H9	29.43	28.17	0.92	0.42	H165	0.00	25.51	0.48	0.14
O82	29.24	10.91	0.48	-0.52	C142	20.86	6.63	6.57	0.06	H10	29.51	29.07	1.96	0.42	H166	0.00	29.01	0.43	0.08
O83	25.44	10.28	0.03	-0.56	C143	22.01	6.19	7.23	-0.12	H11	1.82	9.48	5.63	0.11	H167	0.00	23.33	4.52	0.08
O84	25.95	7.22	9.01	-0.78	C144	19.62	6.23	7.32	0.20	H12	3.65	8.42	2.18	0.11	H168	0.00	27.23	4.67	0.13
O85	9.23	27.30	1.69	-0.77	C145	2.61	27.36	5.14	-0.12	H13	5.69	8.42	3.26	0.11	H169	7.18	0.00	8.40	0.14
O86	9.44	24.76	9.57	-0.58	C146	2.51	27.68	3.79	0.05	H14	3.94	9.83	9.63	0.12	H170	10.69	0.00	8.35	0.08
O87	9.70	24.73	1.54	-0.58	C147	3.69	28.01	3.11	-0.11	H15	5.73	12.12	1.93	0.13	H171	5.01	0.00	1.88	0.08
O88	7.41	25.74	0.48	-0.52	C148	6.25	27.83	5.82	0.62	H16	1.85	10.47	10.34	0.11	H172	8.91	0.00	2.03	0.13
O89	11.21	26.37	0.03	-0.56	C149	5.09	25.79	0.41	-0.03	H17	3.88	9.60	6.69	0.12	H173	25.24	18.32	2.91	0.14
O90	10.70	29.43	9.01	-0.78	C150	4.97	27.69	5.11	-0.01	H18	3.60	12.69	2.71	0.12	H174	29.01	18.32	3.07	0.08
O91	27.55	8.98	6.97	-0.77	C151	6.41	26.17	10.51	0.62	H19	11.11	9.84	6.20	0.42	H175	23.26	18.32	7.84	0.14
O92	27.76	6.43	4.29	-0.58	C152	4.90	28.01	3.74	-0.08	H20	11.19	10.75	7.23	0.42	H176	27.23	18.32	7.31	0.13
O93	28.03	6.40	6.82	-0.58	C153	3.88	26.23	10.36	-0.08	H21	34.82	27.17	5.63	0.11	H177	11.55	18.32	2.84	0.14
O94	25.74	7.41	5.76	-0.52	C154	1.20	27.81	3.14	0.23	H22	33.00	28.23	2.18	0.11	H178	7.64	18.32	3.07	0.08
O95	29.53	8.04	5.31	-0.56	C155	4.96	24.88	1.51	-0.08	H23	30.96	28.23	3.26	0.11	H179	13.35	18.33	7.84	0.14
O96	29.02	11.11	3.73	-0.78	C156	2.63	25.84	0.22	-0.13	H24	32.71	26.82	9.63	0.12	H180	9.42	18.32	7.31	0.13
C1	20.94	27.61	10.42	-0.12	C157	3.83	27.32	5.78	-0.10	H25	30.92	24.53	1.93	0.13	H181	29.47	0.00	8.40	0.14
C2	20.83	27.29	9.07	0.05	C158	2.54	24.95	1.29	0.06	H26	34.80	26.18	10.34	0.11	H182	25.96	0.00	8.35	0.08
C3	22.01	26.97	8.38	-0.11	C159	3.69	24.51	1.95	-0.12	H27	32.77	27.05	6.69	0.12	H183	31.64	0.00	1.88	0.08
C4	24.58	27.14	0.55	0.62	C160	1.20	24.50	1.79	0.20	H28	33.04	23.95	2.71	0.12	H184	27.74	0.00	2.03	0.13

C5	23.41	29.18	5.68	-0.03	C161	34.03	9.28	5.14	-0.12	H29	25.54	26.80	6.20	0.42	H185	18.32	6.86	5.53	0.13
C6	23.29	27.28	10.39	-0.01	C162	34.14	8.96	3.79	0.05	H30	25.46	25.90	7.23	0.42	H186	18.32	10.69	5.71	0.08
C7	24.74	28.80	5.23	0.62	C163	32.96	8.64	3.11	-0.11	H31	16.50	8.84	0.35	0.11	H187	18.32	4.95	10.48	0.14
C8	23.22	26.96	9.02	-0.08	C164	30.40	8.82	5.82	0.62	H32	14.67	9.90	7.46	0.11	H188	18.32	8.91	9.95	0.13
C9	22.20	28.75	5.09	-0.08	C165	31.56	10.86	0.41	-0.03	H33	12.63	9.90	8.54	0.11	H189	0.00	11.14	0.48	0.14
C10	19.53	27.16	8.42	0.23	C166	31.68	8.96	5.11	-0.01	H34	14.38	8.50	4.35	0.12	H190	0.00	7.64	0.43	0.08
C11	23.28	30.09	6.78	-0.08	C167	30.23	10.48	10.51	0.62	H35	12.60	6.21	7.21	0.13	H191	0.00	13.31	4.52	0.08
C12	20.96	29.13	5.50	-0.13	C168	31.75	8.64	3.74	-0.08	H36	16.47	7.85	5.07	0.11	H192	0.00	9.42	4.67	0.13

Supplementary Table 9. The simulated parameters of benzene and toluene.

# Benzene				# Toluene			
# number of atoms				# number of atoms			
12				12			
# atomic positions				# atomic positions			
0 C_benz	0.00000000	0.00000000	0.00000000	0 C_xyl	0.00000000	0.00000000	0.00000000
1 C_benz	1.39200000	0.00000000	0.00000000	1 C_xyl	1.40000000	0.00000000	0.00000000
2 C_benz	2.08800000	1.20550736	0.00000000	2 C_xyl	2.10000000	1.21243557	0.00000000
3 C_benz	1.39200051	2.41101443	-0.00119599	3 C_xyl	1.40000000	2.42487113	0.00000000
4 C_benz	0.00000000	2.41101459	-0.00167420	4 C_xyl	-0.00000000	2.42487113	0.00000000
5 C_benz	-0.69600000	1.20550717	-0.00068061	5 C_xyl	-0.70000000	1.21243557	0.00000000
6 H_benz	-0.54000000	-0.93530733	0.00044182	6 CH3_xyl	-0.755000	-1.30769836	0.00000000
7 H_benz	1.93200000	-0.93530654	0.00129125	7 H_xyl	1.94000000	-0.93530744	0.00000000
8 H_benz	3.16799982	1.20550747	0.00062273	8 H_xyl	3.18000000	1.21243557	-0.00000000
9 H_benz	1.93200056	3.34632183	-0.00125416	9 H_xyl	1.94000000	3.36017857	0.00000000
10 H_benz	-0.53999901	3.34632180	-0.00260212	10 H_xyl	-0.5400000	3.36017857	0.00000000
11 H_benz	-1.77600000	1.20550708	-0.00085731	11 H_xyl	-1.7800000	1.21243557	0.00000000

Supplementary Table 10. Fitting parameters of DSLF model for benzene and cyclohexane adsorption isotherms fitting on ZJU-520(Al) at 298 K.

Parameters	Benzene	Cyclohexane
Q_{m1} (mmol g^{-1})	5.01	16.58
b_1 (kPa^{-1})	64691.33	0.060
n	5.35	0.33
Q_{m2} (mmol g^{-1})	5.011	3.28
b_2 (kPa^{-1})	64691.33	3257771.90
m	5.35	5.69
R^2	0.96	0.99

Supplementary References

1. Pei J*, et al.* Dense packing of acetylene in a stable and low-cost metal-organic framework for efficient C_2H_2/CO_2 separation. *Angewandte Chemie International Edition* **60**, 25068 – 25074 (2021).
2. Burtch NC*, et al.* Water stability and adsorption in metal-organic frameworks. *Chemical Reviews* **114**, 10575 – 10612 (2014).
3. Maddox MW*, et al.* Characterization of MCM-41 using molecular simulation: Heterogeneity effects. *Langmuir* **13**, 1737 – 1745 (1997).
4. Gong L*, et al.* A superhydrophobic and porous polymer adsorbent with large surface area. *Journal of Materials Chemistry A* **9**, 254 – 258 (2021).
5. Yang K*, et al.* Adsorption of volatile organic compounds by metal-organic frameworks MIL-101: Influence of molecular size and shape. *Journal of Hazardous materials* **195**, 124 – 131 (2011).
6. Gao J*, et al.* A microporous hydrogen-bonded organic framework for the efficient capture and purification of propylene. *Angewandte Chemie International Edition* **60**, 20400 – 20406 (2021).
7. Gu XW*, et al.* Immobilization of lewis basic sites into a stable ethane-selective MOF enabling one-step separation of ethylene from a ternary mixture. *Journal of the American Chemical Society* **144**, 2614 – 2623 (2022).
8. Serafin J*, et al.* The new tailored nanoporous carbons from the common polypody (Polypodium vulgare): The role of textural properties for enhanced CO_2 adsorption. *Chemical Engineering Journal* **429**, 131751 – 131756 (2022).
9. Toby BH, Von Dreele RB. GSAS-II: The genesis of a modern open-source all purpose crystallography software package. *Journal of Applied Crystallography* **46**, 544 – 549 (2013).
10. Burla MC*, et al.* SIR2004: An improved tool for crystal structure determination and refinement. *Journal of Applied Crystallography* **38**, 381 – 388 (2010).
11. Gandara F*, et al.* High methane storage capacity in aluminum metal-organic

frameworks. *Journal of the American Chemical Society* **136**, 5271 – 5280 (2014).

12. Sheldrick GM. Shelxt-integrated space-group and crystal-structure determination. *Acta Crystallographica Section A: Foundations and Advances* **71**, 3-8 (2015).

13. Sheldrick GM. Crystal structure refinement with SHELXL. *Acta Crystallographica Section C-Structural Chemistry* **71**, 3 – 8 (2015).

14. Dolomanov OV*, et al.* Olex2: A complete structure solution, refinement and analysis program. *Journal of Applied Crystallography* **42**, 339 – 341 (2009).

15. Chen Z*, et al.* Reticular access to highly porous acs-MOFs with rigid trigonal prismatic linkers for water sorption. *Journal of the American Chemical Society* **141**, 2900 – 2905 (2019).

16. Spek AL. Structure validation in chemical crystallography. *Acta Crystallographica Section D-Structural Biology* **65**, 148 – 155 (2009).

17. Dubbeldam D*, et al.* RASPA: Molecular simulation software for adsorption and diffusion in flexible nanoporous materials. *Molecular Simulation* **42**, 81 – 101 (2016).

18. Widom B. Some topics in the theory of fluids. *The Journal of Chemical Physics* **39**, 2808 – 2812 (1963).

19. Hu L*, et al.* A novel aluminum-based metal-organic framework with uniform micropores for trace BTEX adsorption. *Angewandte Chemie International Edition* **62**, e202215296 (2023).

20. Stöhr M*, et al.* Theory and practice of modeling van der Waals interactions in electronic-structure calculations. *Chemical Society Reviews* **48**, 4118 – 4154 (2019).

21. Grudinin S, Redon S. Practical modeling of molecular systems with symmetries. *Journal of Computational Chemistry* **31**, 1799 – 1814 (2010).

22. Manz TA, Limas NG. Introducing DDEC6 atomic population analysis: Part 1.

Charge partitioning theory and methodology. *RSC Advances* **6**, 47771 – 47801 (2016).

23. Manz TA, Sholl DS. Chemically meaningful atomic charges that reproduce the electrostatic potential in periodic and nonperiodic materials. *Journal of Chemical Theory and Computation* **6**, 2455 – 2468 (2010).

24. Gong W*, et al.* Wicking-polarization-induced water cluster size effect on triboelectric evaporation textiles. *Advanced Materials* **33**, 2007352 – 2007363 (2021).

25. Zhang C*, et al.* Enantiomeric MOF crystals using helical channels as palettes with bright white circularly polarized luminescence. *Advanced Materials* **32**, 2002914 – 2002923 (2020).

26. Rappe AK*, et al.* UFF, a full periodic table force field for molecular mechanics and molecular dynamics simulations. *Journal of the American Chemical Society* **114**, 10024 – 10035 (1992).

27. Sasaki K, Yamashita T. Modification and validation of the Dreiding force field for molecular liquid simulations (Dreiding-UT). *Journal of Chemical Information and Modeling* **61**, 1172 – 1179 (2021).

28. Casewit CJ*, et al.* Application of a universal force field to organic molecules. *Journal of the American Chemical Society* **114**, 10035 – 10046 (1992).

29. Pang J*, et al.* A porous metal-organic framework with ultrahigh acetylene uptake capacity under ambient conditions. *Nature Communications* **6**, 7575 – 7580 (2015).

30. Yu X*, et al.* Determination of CH_4, C_2H_6 and CO_2 adsorption in shale kerogens coupling sorption-induced swelling. *Chemical Engineering Journal* **410**, 127690 – 127702 (2021).

31. Mukherjee S*, et al.* Harnessing Lewis acidic open metal sites of metal-organic frameworks: the foremost route to achieve highly selective benzene sorption over cyclohexane. *Chemical Communications* **52**, 8215 – 8218 (2016).

32. Kühne TD*, et al.* CP2K: An electronic structure and molecular dynamics software package-quickstep: Efficient and accurate electronic structure calculations. *The Journal of Chemical Physics* **152**, 7534 – 7542 (2020).

33. VandeVondele J*, et al.* Quickstep: Fast and accurate density functional calculations using a mixed Gaussian and plane waves approach. *Computer Physics Communications* **167**, 103 – 128 (2005).

34. Lu T, Chen F. Multiwfn: A multifunctional wavefunction analyzer. *Journal of Computational Chemistry* **33**, 580 – 592 (2012).

35. Ernzerhof M, Scuseria GE. Assessment of the Perdew-Burke-Ernzerhof exchange-correlation functional. *The Journal of Chemical Physics* **110**, 5029 – 5036 (1999).

36. Paier J*, et al.* The Perdew-Burke-Ernzerhof exchange-correlation functional applied to the G2-1 test set using a plane-wave basis set. *The Journal of Chemical Physics* **122**, 234102 – 234112 (2005).

37. Perdew JP*, et al.* Generalized gradient approximation made simple. *Physical Review Letters* **77**, 3865 – 3868 (1996).

38. He T*, et al.* Trace removal of benzene vapour using double-walled metal-dipyrazolate frameworks. *Nature Materials* **21**, 689 – 695 (2022).

39. Mo Q*, et al.* Engineering single-atom sites into pore-confined nanospaces of porphyrinic metal-organic frameworks for the highly efficient photocatalytic hydrogen evolution reaction. *Journal of the American Chemical Society* **144**, 22747 – 22758 (2022).

40. Mileo PGM*, et al.* Achieving superprotonic conduction with a 2D fluorinated metal-organic framework. *Journal of the American Chemical Society* **140**, 13156 – 13160 (2018).

41. Gan L*, et al.* A highly water-stable meta-carborane-based copper metal-organic framework for efficient high-temperature butanol separation. *Journal of the American Chemical Society* **142**, 8299 – 8311 (2020).

42. Stylianou KC, *et al.* Dimensionality transformation through paddlewheel reconfiguration in a flexible and porous Zn-based metal-organic framework. *Journal of the American Chemical Society* **134**, 20466 – 20478 (2012).

43. Goedecker S, *et al.* Separable dual-space Gaussian pseudopotentials. *Physical Review B* **54**, 1703 – 1710 (1996).

44. Pratik SM, *et al.* Engineering electrical conductivity in stable Zirconium-based PCN-222 MOFs with permanent mesoporosity. *Chemistry of Materials* **32**, 6137 – 6149 (2020).

45. Perdew JP, *et al.* Restoring the density-gradient expansion for exchange in solids and surfaces. *Physical Review Letters* **100**, 136406 – 136414 (2008).

46. Wang Y, *et al.* Construction of fluorinated propane-trap in metal-organic frameworks for record polymer-grade propylene production under high humidity conditions. *Advanced Materials* **35**, 2207955 – 2207963 (2023).

47. Meng R, *et al.* Two-dimensional organic-inorganic heterostructures of in situ-grown layered COF on Ti_3C_2 MXene nanosheets for lithium-sulfur batteries. *Nano Today* **35**, 100991 – 100102 (2020).

48. Mukherjee S, *et al.* Advances in adsorptive separation of benzene and cyclohexane by metal-organic framework adsorbents. *Coordination Chemistry Reviews* **437**, 213852 – 213865 (2021).

49. Sapianik AA, *et al.* Exceptionally effective benzene/cyclohexane separation using a nitro-decorated metal-organic framework. *Chemical Communications* **56**, 8241 – 8244 (2020).

50. Xie L, *et al.* Metal-organic frameworks for the capture of trace aromatic volatile organic compounds. *Chem* **4**, 1911 – 1927 (2018).

51. Yang K, *et al.* Adsorption of volatile organic compounds by metal-organic frameworks MOF-177. *Journal of Environmental Chemical Engineering* **1**, 713 – 718 (2013).

52. Zhao Z, *et al.* Competitive adsorption and selectivity of benzene and water

vapor on the microporous metal organic frameworks (HKUST-1). *Chemical Engineering Journal* **259**, 79 – 89 (2015).

53. Jhung SH*, et al.* Microwave synthesis of chromium terephthalate MIL-101 and its benzene sorption ability. *Advanced Materials* **19**, 121 – 124 (2007).

54. Mullangi D*, et al.* Super-hydrophobic covalent organic frameworks for chemical resistant coatings and hydrophobic paper and textile composites. *Journal of Materials Chemistry A* **5**, 8376 – 8384 (2017).

55. Han Y*, et al.* Control of the pore chemistry in metal-organic frameworks for efficient adsorption of benzene and separation of benzene/cyclohexane. *Chem* **9**, 739 – 754 (2023).
