# Supplementary material for: Double-walled Al-based MOF with large microporous specific surface area for trace benzene adsorption
Source: Nat Commun. 2024 Apr 13;15:3204. doi: 10.1038/s41467-024-47612-x (PMC11016061; doi:10.1038/s41467-024-47612-x)
Supplement: Supplementary file 3 — Description of Additional Supplementary Files [file 41467_2024_47612_MOESM3_ESM.pdf]

Description of Additional Supplementary Files

Supplementary Movie 1

Description: The process of benzene adsorption by ZJU-520(Al).

Supplementary Movie 2

Description: The process of toluene adsorption by ZJU-520(Al).
